# Intramolecular Nicholas Reaction Enables the Stereoselective Synthesis of Strained Cyclooctynes

**DOI:** 10.3390/molecules26061629

**Published:** 2021-03-15

**Authors:** Diego M. Monzón, Juan Manuel Betancort, Tomás Martín, Miguel Ángel Ramírez, Víctor S. Martín, David Díaz Díaz

**Affiliations:** 1 Departamento de Química Orgánica, Universidad de La Laguna, Avda. Astrofísico Francisco Sánchez 3, 38206 La Laguna, Tenerife, Spain; dmonzon@ull.edu.es (D.M.M.); mramirez@ull.edu.es (M.Á.R.); 2 Instituto de Bio-Orgánica Antonio González, Universidad de La Laguna, Avda. Astrofísico Francisco Sánchez 3, 38206 La Laguna, Tenerife, Spain; jmapt.8537@gmail.com (J.M.B.); tmartin@ipna.csic.es (T.M.); 3 Instituto de Productos Naturales y Agrobiología, CSIC, Francisco Sánchez 3, 38206 La Laguna, Tenerife, Spain; 4 Institut für Organische Chemie, Universität Regensburg, Universitätsstr. 31, 93053 Regensburg, Germany

**Keywords:** Nicholas reaction, cyclooctyne, propargylic carbocation, cyclization, oxacycle, ring strain

## Abstract

Cyclic products can be obtained through the intramolecular version of the Nicholas reaction, which requires having the nucleophile connected to the alkyne unit. Here, we report the synthesis of 1-oxa-3-cyclooctynes starting from commercially available (1*R*,3*S*)-camphoric acid. The strategy is based on the initial preparation of propargylic alcohols, complexation of the triple bond with Co_2_(CO)_8_, and treatment with BF_3_·Et_2_O to induce an intramolecular Nicholas reaction with the free hydroxyl group as nucleophile. Finally, oxidative deprotection of the alkyne afforded the cyclooctynes in good yields. Notably, large-sized R substituents at the chiral center connected to the O atom were oriented in such a way that steric interactions were minimized in the cyclization, allowing the formation of cyclooctynes exclusively with *(R)* configuration, in good agreement with theoretical predictions. Moreover, preliminary studies demonstrated that these cyclooctynes were reactive in the presence of azides yielding substituted triazoles.

## 1. Introduction

Organometallic complexes have found extensive use in organic chemistry due to the unique properties they impart on the coordinated organic ligands. A notable transformation that showcases this altered reactivity is the Nicholas reaction [1,2,3,4,5,6]. It involves the reaction of dicobalt hexacarbonyl-stabilized propargylic carbocations with nucleophiles. The remarkable stability of these carbocations arises from delocalization of the cationic charge onto the metallic complex. The mild conditions used to form the acetylenic cobalt complexes, coupled with the versatility of the highly functionalized reaction products obtained upon demetallation, has rendered this reaction a useful tool for the synthesis of diverse and complex molecules [7,8,9,10,11].

An extensive list of nucleophiles has been employed in this transformation, from carbon-based entities such as ketones and enol silanes, to nitrogen nucleophiles such as amines and sulfonamides, or oxygen centered species such as alcohols [12,13] or epoxides [14,15]. In the case where the nucleophile is connected to the alkyne via a suitable chain, the Nicholas reaction will result in intramolecular attack and formation of a ring [12,13]. This process is favored by the altered geometry of the complexed triple bond that reduces ring strain. Of particular interest are cyclooctynes, the smallest stable cyclic alkynes. Their inherent strain has been exploited in organic synthesis and bioorthogonal chemistry for the development of novel cycloaddition reactions and labeling agents [16,17,18]. Introduction of heteroatoms in their structure has subtle effects on the reactivity and stability of the resulting rings [19]. Herein, we describe the synthesis of 1-oxa-3-cyclooctyne derivatives by making use of an intramolecular Nicholas reaction. The use of this reaction has been also reported to obtain other strained cycloalkynes such as cyclononynes [20].

## 2. Results and Discussion

**Scheme 1:**
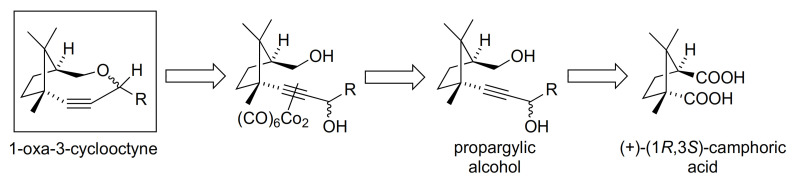
Retrosynthetic analysis of the cyclooctynes envisioned in this work.

Our synthetic design towards the preparation of strained 1-oxa-3-cyclooctynes via the Nicholas reaction was based on the use of (1*R*,3*S*)-camphoric acid as starting material (Scheme 1), a low-cost natural product that is commercially available in both enantiomeric forms. Apart from the cyclopentane ring, the structure of the desired cyclooctynes is characterized by the presence of two quaternary centers at α- and β-positions of the triple bond, thus providing a sterically demanding environment around it. 

In this way, we first envisioned the preparation of the corresponding propargylic alcohols as intermediate products, which could be obtained from (+)-(1*R*,3*S*)-camphoric acid. The camphoric acid not only provides a cyclic structure to contribute to the preorganization of the system prior to cyclization, but it also enables the presence of the oxygen heteroatom, which would act as the nucleophile during formation of the oxacycle.

**Scheme 2:**
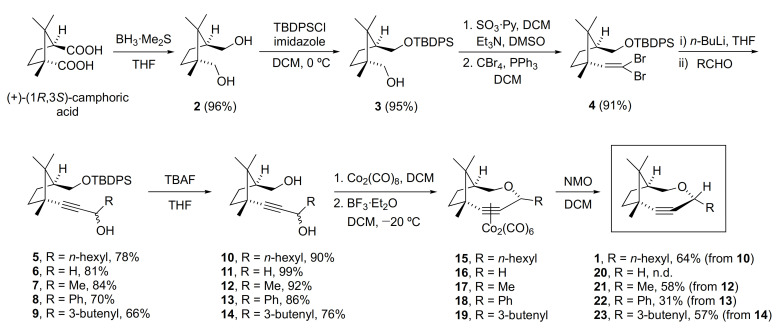
Synthetic route for the preparation of strained cyclooctynes (**1**, **20**−**23**) starting from commercially available (+)-(1*R*,3*S*)-camphoric acid. Abbreviation: n.d. = not detected.

Therefore, the first objective was to form a suitable precursor of the propargylic alcohol, from which we could introduce different R substituents. For this purpose, we began with the reduction of (+)-(1*R*,3*S*)-camphoric acid using borane complexes to obtain the corresponding diol **2** (Scheme 2). In this step, the best result was obtained with the BH_3_·SMe_2_ complex in THF, affording the desired diol **2** in 96% yield. Subsequently, the different steric demand of both alcohols in **2**, dictated by the presence of the chiral methyl group, made it possible to perform the selective protection of the most accessible hydroxyl group as its *t*-butyldiphenylsilyl ether at 0 °C in 1 h, affording product **3** in excellent yield (95%).

The next step required the transformation of the free hydroxyl group into a terminal alkyne, for which the introduction of an additional carbon atom was necessary. This was satisfactorily achieved using the well-known Corey–Fuchs reaction [21]. Firstly, the oxidation of the primary hydroxyl group of compound **3** was carried out using the Parikh–Doering reaction [22], affording the corresponding aldehyde. Then, the so-formed aldehyde was reacted with a mixture of PPh_3_ and CBr_4_ to give in good yield (91%) the expected 1,1-dibromoalkene derivative **4** [23]. At this point, an excess of *n-*BuLi at −78 °C was added to compound **4** to form the corresponding alkanide anions, which were subsequently reacted with different aldehydes (i.e., heptanal, *para*-formaldehyde, acetaldehyde, benzaldehyde, 4-pentenal). This resulted in the formation of a series of propargylic alcohols **5**–**9** in good yields (66–84%) with different R substituents (i.e., R = hexyl, R = H, R = Me, R = Ph, R = butenyl, respectively). Removal of the *t*-butyldiphenylsilyl ether group (TBDPS) using tetra-*n*-butylammonium fluoride in THF yielded the desired diols **10**–**14** in good yields (76–99%), which represent the starting materials for testing the intramolecular Nicholas reaction.

Provided an optimal interatomic distance, the nucleophilic free hydroxyl group should attack at the propargylic position, necessary in order to obtain the desired cyclooctynes [3]. Cyclization was induced by forming a stabilized carbocation upon complexation of the triple bond with dicobalt octacarbonyl [Co_2_(CO)_8_] and subsequent treatment with a Lewis acid. Thus, bright red-colored cobalt complexes were easily obtained at room temperature in DCM in almost quantitative yields upon a quick purification by column chromatography. It is worth mentioning that the cobalt complexes were always prepared immediately prior to their use, even though they were stable for several days under inert atmosphere at −20 °C. Long periods of time, even under these conditions, caused their slow but gradual decomposition. Finally, the obtained cobalt complexes were re-dissolved in DCM and treated with a slight excess of BF_3_·Et_2_O (1.2 equiv) at −20 °C. This prompted the intramolecular Nicholas reaction to afford the corresponding cyclic ethers **15**–**19**, which were immediately treated with 4-methylmorpholine *N*-oxide (NMO) as a mild oxidizer to remove the cobalt complexation of the triple bonds. The strained cycloalkynes **1** and **21**–**23** were isolated in good overall yields after three steps (31–64%). However, the expected product **20** (R = H) could not be detected from the reaction mixture obtained from precursor **11**. Further investigation will be carried out in our group to clarify this unexpected result.

Mono- and bidimensional NMR spectroscopy confirmed unequivocally the structures of all synthesized compounds **1** and **21**–**23** (Figures S1–S46). Very interestingly, a series of ROESY experiments showed that the stereochemical configuration of the chiral center generated during cyclization was exclusively (*R*) for compounds **1**, **22**, and **23** (Figure 1). 

**Scheme 3:**
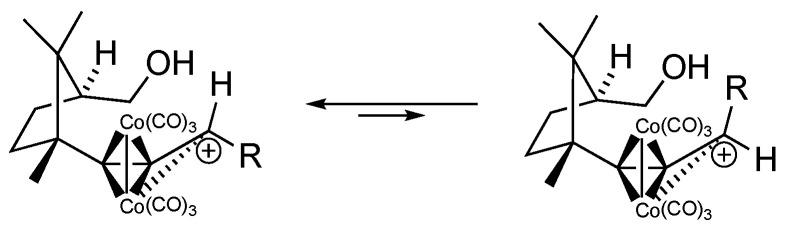
The generation of (*S*) configuration at the stabilized carbocation in the cyclization is hindered by steric factors in the case of large-sized R groups.

The observed stereoselectivity can be explained on the basis of the reaction mechanism because the orientation of the substituents is fixed during the generation of the intermediate carbocation and it is defined by steric factors (Scheme 3). In particular, large-sized R substituents (e.g., R = *n*-hexyl, Ph, 3-butenyl) are oriented in such a way that they can minimize steric interactions, as observed in the case of compounds **1**, **22**, and **23**. In contrast, when R substituents are of small size (e.g., R = Me) the steric interactions are smaller, and the free rotation of the bond between the cobalt-bonded carbon and the carbon at the propargylic position allows for the substituent to be oriented in any direction and, hence, the intramolecular attack occurs on both sides of the carbocation. This justifies the formation of the two possible stereoisomers (*R*) and (*S*) in the case of compound **21**. Therefore, the side preference during the intermolecular nucleophilic attack on the carbocation depends on both the nature of the propargylic alcohol and the size of the R substituent.

**Figure 2:**
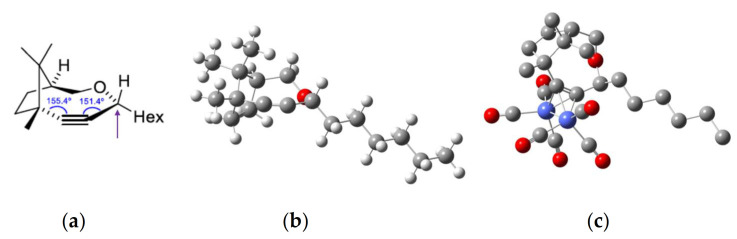
(**a**) Calculated angle-strain in cyclooctyne **1**; (**b**) 3D ball-and-stick molecular model of minimized (*R*)-**1**; (**c**) 3D ball-and-stick molecular model of minimized (*R*)-**15** (hydrogen atoms are omitted for clarity).

Furthermore, these results were found to be in good agreement with theoretical calculations. Specifically, the structural analysis of the cycloalkyne **1** was performed at the B3LYP/6-31G* level to determine the bond angle between the carbon atoms of the triple bond and the neighboring carbons and the stability of both stereoisomers (i.e., (*R*) and (*S*) at the chiral center connected to the O atom). The results showed that the deviation from linearity at the triple bond was 53.2°, corresponding to two sp^3^-sp angles of 155.4 and 151.4° for (*R*)-**1** (left and right of the image, respectively, Figure 2a), and 155.8 and 155.4° for the isomer (*S*)-**1**.

Furthermore, the stability of compound **1**, which has an (*R*) configuration at the chiral center (marked with an arrow in Figure 2a) was 1.51 kcal/mol higher than its analog with (*S*) configuration. In addition, calculations performed for the cobalt-complex precursor **15**, with B3LYP/def2SVP optimization, revealed that such difference in stability increased to 7.62 kcal/mol. Thus, the product with *(R)* configuration is favored enthalpically, as is the formation of the cobalt complex precursor. Clearly, the angular stress release as a consequence of complexation favors the Nicholas reaction, being that its selectivity is controlled by the electrostatic and steric effects generated in the new scenario.

**Scheme 4:**
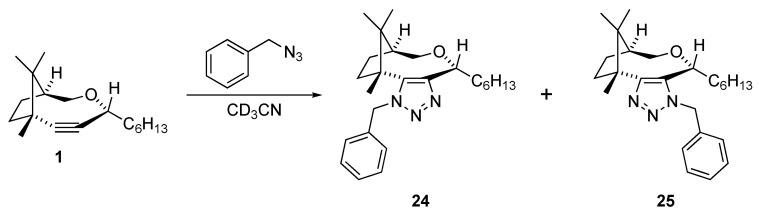
Strain-promoted azide-alkyne cycloaddition (SPAAC) between synthesized cyclooctyne **1** and benzylazide.

Finally, a simple preliminary test was carried out in order to evaluate the potential use of these compounds in the strain-promoted azide-alkyne cycloaddition (SPAAC) [17]. In this experiment, a solution of benzyl azide in CD_3_CN was added to a solution of **1** in CD_3_CN, in a 1:1 ratio (azide/cycloalkyne) (Scheme 4), and the reaction was monitored over time by ^1^H-NMR spectroscopy at 25 °C (Figure S47). The analysis of the spectra clearly showed the disappearance of the signals ascribed to the protons in the benzylic position of the benzyl azide as well as the appearance of new benzylic protons associated to the formation of the corresponding triazole rings (**24** and **25**). However, despite the large size of the bicyclic structure, no regioselectivity was detected and both regioisomers were obtained in almost the same proportion. In terms of kinetics, the second-order rate constant was estimated in 7.6 × 10^−3^ M^−1^s^−1^, which is in the range of other cyclooctynes [24].

It is worth noting that other strained cyclooctynes such as **23**, bearing a terminal double bond, may also expand the potential uses of these compounds since further functionalization can be achieved through another “click” reaction such as the thiol-ene reaction [25].

## 3. Materials and Methods

### 3.1. Instrumental Techniques

- Nuclear magnetic resonance (NMR)

^1^H-NMR spectra were recorded in BRUKER AVANCE spectrometers (Bruker Corporation, Billerica, MA, USA) at 500 and 600 MHz, and ^13^C-NMR spectra were recorded at 125 and 150 MHz. Chemical shifts were reported in units (ppm) by assigning tetramethylsilane resonance in the ^1^H-NMR spectrum as 0.00 ppm (chloroform, 7.26 ppm). Data were reported as follows: chemical shift, multiplicity coupling constant (*J* values) in Hz and integration. Chemical shifts for ^13^C-NMR spectra were recorded in ppm from tetramethylsilane using the central peak of CDCl_3_ (77.0 ppm) as the internal standard. In ^1^H-NMR spectra, multiplicity is indicated by the following abbreviations: singlet = s, doublet = d, triplet = t, quartet = q, double doublet = dd, double double doublet = ddd, multiplet = m. In ^13^C-NMR spectra, multiplicity is indicated as following: CH_3_ = q, CH_2_ = t, CH = d, C = s. Copies of all NMR spectra are included as Supplementary Material in Appendix A.

- Mass spectrometry

Accurate mass (HRMS) was determined by electrospray ionization (ESI-TOF) and electronic impact (EI-TOF).

- Melting point

Melting point was measured by a Büchi B-540 equipment (BUCHI corporation, New Castle, DE, USA), using a Standard vanillin sample (Büchi Nº 37454) with a melting point value of 81.7 °C ± 0.7 °C as a reference standard.

- Specific rotation (polarimetry)

Specific rotation measurements were carried out at 25 °C in a Perkin-Elmer 241 (PerkinElmer Corporation, Waltham, MA, USA) polarimeter equipped with a Na lamp. The concentration of the samples was indicated in each case using 1 dm long cells.

### 3.2. Chromatographic Techniques

Flash column chromatography was performed using silica gel, 60 Å and 0.2–0.5 mm, with the indicated solvent system according to standard techniques. Compounds were visualized on TLC plates by use of UV light, or vanillin with acetic and sulfuric acid in ethanol with heating.

### 3.3. Solvents and Reagents

DCM, Et_2_O, and THF were dried and distilled under argon atmosphere immediately prior to use. Other commercially available solvents properly stored were used without further purification. All reagents were purchased from commercial suppliers and used without further purification, unless otherwise stated.

### 3.4. Nomenclature

Chemical nomenclature was generated using ChemDraw Professional version 17.1.0.105 (19) software (PerkinElmer Corporation, Massachusetts, USA), and in accordance with IUPAC rules. The atoms of the compounds were numbered following the abovementioned nomenclature, unless noted otherwise.

### 3.5. Synthesis and Characterization of Compounds

#### 3.5.1. *((1R,3S)-1,2,2-trimethylcyclopentane-1,3-diyl)dimethanol* (**2**) 

To a solution of camphoric acid (7.1 g, 35.0 mmol) in 250 mL THF at 0 °C under inert atmosphere, we added 46 mL of BH_3_·SMe_2_ (2.0 M in THF) dropwise, and the reaction mixture was stirred for 23 h at RT. The reaction was quenched by adding 17 mL of MeOH dropwise at 0 °C. Then, the solvent was evaporated under reduced pressure. The crude was purified by flash chromatography (silica gel, gradient *n*-hexane/EtOAc 60:40 to 40:60) to obtain 5.8 g (96% yield) of **2** as a white solid.

^1^H-NMR (500 MHz, CDCl_3_, 298 K): δ ppm 3.73 (dd, *J* = 5.3, 10.3 Hz, 1H), 3.58 (d, *J* = 10.8 Hz, 1H), 3.51 (dd, *J* = 8.3, 10.3 Hz, 1H), 3.47 (d, *J* = 10.8 Hz, 1H), 2.08 (ddd, *J* = 5.3, 9.3, 17.8 Hz, 1H), 1.99–1.91 (m, 1H), 1.63–1.56 (m, 1H), 1.41–1.32 (m, 2H), 1.25 (br, 2H), 1.02 (s, 3H), 1.01 (s, 3H), 0.79 (s, 3H).

^13^C-NMR (125 MHz, CDCl_3_, 298 K): δ ppm 69.2 (t), 65.0 (t), 50.5 (d), 48.8 (s), 44.0 (s), 33.7 (t), 25.5 (t), 24.2 (q), 20.4 (q), 18.5 (q).

HRMS (ESI-negative): *m/z*: calculated for C_10_H_19_O [M − H^+^]: 171.1385, found: 171.1391.

Melting point: 130–132 °C.

[α]^25^_D_ = + 54.1 (*c* 2.19, CHCl_3_).

#### 3.5.2. *((1R,3S)-3-(((tert-butyldiphenylsilyl)oxy)methyl)-1,2,2-trimethylcyclopentyl)methanol* (**3**)

The diol **2** (8.4 g, 48.8 mmol) was dissolved in 500 mL of DCM (0.1 M) under inert atmosphere (Ar). The solution was then cooled to 0 °C and imidazole (10.0 g, 146.3 mmol, 3 equiv) was added. Finally, TBDPSCl (13 mL, 48.8 mmol, 1.0 equiv) was added dropwise and the mixture was stirred for 3–4 h. The reaction was quenched with 800 mL of water and the 2 phases were separated. The aqueous layer was extracted with DCM (2 × 400 mL). The organic layers were collected, dried over anhydrous MgSO_4_, and filtered, and the solvent was removed under reduced pressure. The crude was purified by flash chromatography (silica gel, gradient *n*-hexane/EtOAc, 90:10 to 80:20) to obtain 19.1 g (95% yield) of the compound **3** as a colorless oil.

^1^H-NMR (500 MHz, CDCl_3_, 298 K): δ ppm 7.69–7.66 (m, 4H), 7.45–7.36 (m, 6H), 3.70 (dd, *J* = 6.4, 10.1 Hz, 1H), 3.57–3.52 (m, 2H), 3.45 (d, *J* = 10.8 Hz, 1H), 2.14 (ddd, *J* = 7.2, 9.6, 16.4 Hz, 1H), 1.92–1.83 (m, 1H), 1.58–1.50 (m, 1H), 1.36–1.24 (m, 2H), 1.04 (s, 9H), 0.99 (s, 3H), 0.97 (s, 3H), 0.75 (s, 3H).

^13^C-NMR (150 MHz, CDCl_3_, 298 K): δ ppm 135.6 (d), 134.1 (s), 134.0 (s), 129.5 (d), 129.5 (d), 127.6 (d), 69.3 (t), 65.5 (t), 50.1 (d), 48.9 (s), 43.9 (s), 33.7 (t), 26.9 (q), 25.2 (t), 24.4 (q), 20.4 (q), 19.2 (s), 18.4 (q).

HRMS (ESI): *m/z*: calculated for C_26_H_38_O_2_Si [M + Na^+^]: 433.2539, found: 433.2536

[α]^25^_D_ = + 20.5 (*c* 2.12, CHCl_3_).

#### 3.5.3. *tert-Butyl(((1S,3S)-3-(2,2-dibromovinyl)-2,2,3-trimethylcyclopentyl)methoxy)diphenylsilane* (**4**)

To a solution of **3** (10.1 g, 24.59 mmol) in 82 mL of DCM (0.3 M) under inert atmosphere (Ar) at 0 °C, we sequentially added DMSO (16.2 mL, 0.66 mL/mmol) triethylamine (17.3 mL, 122.97 mmol, 5 equiv) and finally the SO_3_·Py complex (11.7 g, 3 equiv). The mixture was stirred for 2 h. After that, the reaction was quenched with 100 mL of water, and then the organic layer was separated and the aqueous phase was extracted with DCM (2 × 100 mL). The organic extracts were collected, dried over anhydrous MgSO_4_, and filtered, and the solvent was evaporated under reduced pressure. The crude was purified by flash chromatography (silica gel, *n*-hexane/EtOAc, 95:5) to obtain 9.3 g (93% yield) of the corresponding aldehyde, which was immediately used in the next step.

Intermediate aldehyde: ^1^H-NMR (500 MHz, CDCl_3_, 298 K): δ ppm 9.63 (s, 1H) 7.68–7.65 (m, 4H), 7.45–7.37 (m, 6H), 3.6 (dd, *J* = 6.7, 10.2 Hz, 1H), 3.57 (dd, *J* = 6.9, 10.2 Hz, 1H), 2.29 (ddd, *J* = 5.8, 11.5, 13.2, Hz, 1H), 2.15 (ddd, *J* = 6.9, 9.3, 16.3 Hz, 1H), (dddd, *J* = 6.1, 9.5, 13.6, 13.6 Hz, 1H), 1.92–1.83 (m, 1H), 1.45–1.29 (m, 2H), 1.07 (s, 3H), 1.05 (s, 12H), 0.82 (s, 3H).

To a solution of the so-formed aldehyde (1.73 g, 4.23 mmol) in DCM (42.3 mL, 0.1 M) at 0 °C under inert atmosphere (Ar), we added 7.8 g of PPh_3_, and when it was dissolved, 5.0 g of CBr_4_ was added in portions. The mixture was stirred overnight. Next, the solvent was removed under reduced pressure until one-quarter of the volume remained. Then, 50 mL of Et_2_O was added and the suspension was filtered through celite. Finally, the solvent from the filtrate was removed in vacuo. The crude was purified by flash chromatography (silica gel, *n*-hexane/DCM, 98:2) to obtain 2.17 g (91% yield) of the compound **4**.

^1^H-NMR (500 MHz, CDCl_3_, 298 K): δ ppm 7.69–7.65 (m, 4H), 7.45–7.36 (m, 6H). 6.61 (s, 1H), 3.69 (dd, *J* = 6.9, 10.1 Hz, 1H), 3.56 (dd, *J* = 7.0, 10.1 Hz, 1H), 2.09–1.81 (m, 4H), 1.31–1.23 (m, 1H), 1.06 (s, 3H), 1.05 (s, 9H), 0.94 (s, 3H), 0.70 (s, 3H).

^13^C-NMR (125 MHz, CDCl_3_, 298 K): δ ppm 144.1 (d), 135.6 (d), 133.9 (s), 133.9 (s), 129.6 (d), 129.5 (d), 127.6 (d), 85.3 (s), 65.6, (t), 53.4 (s), 47.4 (d), 45.8 (s), 33.8 (t), 26.9 (q), 24.9 (t), 22.2 (q), 20.9 (q), 19.4 (q), 19.2 (s).

HRMS (ESI): *m/z*: calculated for C_27_H_36_^79^Br^81^BrOSi [M + Na+]: 587.0779, found: 587.0780.

[α]^25^_D_ = + 12.8 (*c* 2.95, CHCl_3_).

#### 3.5.4. Compounds **5**–**9**

General procedure: Compound **4** was dissolved in dry THF (6 mL, 0.15M), and under inert atmosphere (Ar), we added 3 equiv of *n*-BuLi solution (2.3 M in hexane) at 0 °C. The reaction mixture was stirred for 15 min and then the corresponding aldehyde (i.e., heptanal → compound **5**; para-formaldehyde → compound **6**; acetaldehyde → compound **7**; benzaldehyde → compound **8**; 4-pentanal → compound **9**) was added at −78 °C. The mixture was allowed to reach RT while it was stirred overnight. The reaction was quenched by adding 20 mL of saturated NH_4_Cl solution, and then it was extracted with Et_2_O (3 × 20 mL). The organic layer was collected, dried over anhydrous MgSO_4_, filtered, and concentrated under reduced pressure. The crude was purified by flash chromatography (silica gel) yielding the corresponding propargyl alcohol.

*1-((1R,3S)-3-(((tert-butyldiphenylsilyl)oxy)methyl)-1,2,2-trimethylcyclopentyl)non-1-yn-3-ol* (**5**). ^1^H-NMR (600 MHz, CDCl_3_, 298 K): δ ppm 7.68–7.65 (m, 4H), 7.44–7.36 (m, 6H), 4.35 (ddd, *J* = 2.2, 6.5, 6.5 Hz, 1H), 3.71 (dd, *J* = 6.7, 10.0 Hz, 1H), 3.57 (dd, *J* = 7.5, 10.0 Hz, 1H), 2.08–2.02 (m, 1H), 1.97–1.91 (m, 1H), 1.88–1.81 (m, 1H), 1.70–1.59 (m, 2H), 1.34–1.26 (m, 8H), 1.13 (s, 3H), 1.05 (s, 9H), 0.96 (s, 3H), 0.90 (s, 3H), 0.89 (t, *J* = 6.7 Hz, 3H).

^13^C-NMR (150 MHz, CDCl_3_, 298 K): δ ppm 135.6 (d), 134.0 (s), 133.9 (s), 129.5 (d), 129.5 (d), 127.6 (d), 90.9 (s), 83.0 (s), 66.0 (t), 62.8 (d), 48.1 (d), 45.7 (s), 45.3 (s), 45.3 (s), 38.3 (t), 37.5 (t), 37.5 (t), 31.7 (t), 28.9 (t), 26.9 (q), 25.3 (t), 25.2 (t), 24.1 (q), 23.4 (q), 22.5 (t), 20.6 (q), 19.2 (s), 14.0 (q).

Column chromatography: *n*-hexane/EtOAc (95:5).

*3-((1R,3S)-3-(((tert-butyldiphenylsilyl)oxy)methyl)-1,2,2-trimethylcyclopentyl)prop-2-yn-1-ol* (**6**). ^1^H-NMR (500 MHz, CDCl_3_, 298 K): δ ppm 7.69–7.66 (m, 4H), 7.45–7.36 (m, 6H), 4.25 (s, 2H), 3.71 (dd, *J* = 6.7, 10.0 Hz, 1H), 3.57 (dd, *J* = 7.4, 10.0 Hz, 1H), 2.10–2.02 (m, 1H), 1.99–1.91 (m, 1H), 1.90–1.81 (m, 1H), 1.64–1.57 (m, 1H), 1.42 (br, 1H), 1.39–1.30 (m, 1H), 1.14 (s, 3H), 1.05 (s, 9H), 0.97 (s, 3H), 0.91 (s, 3H).

^13^C-NMR (125 MHz, CDCl_3_, 298 K): δ ppm 135.6 (d), 134.0 (s), 133.9 (s), 129.5 (d), 129.5 (d), 127.6 (d), 91.9 (s), 79.9 (s), 66.0 (t), 51.5 (t), 48.1 (d), 45.7 (s), 45.3 (s), 37.4 (t), 26.9 (q), 25.3 (t), 24.0 (q), 23.4 (q), 22.5 (t), 20.6 (q), 19.2 (s).

HRMS (ESI): *m/z*: calculated for C_28_H_38_O_2_Si [M + Na^+^]: 457.2539, found: 457.2535.

Column chromatography: *n*-hexane:EtOAc (80:20).

*4-((1R,3S)-3-(((tert-butyldiphenylsilyl)oxy)methyl)-1,2,2-trimethylcyclopentyl)but-3-yn-2-ol* (**7**). ^1^H-NMR (600 MHz, CDCl_3_, 298 K): δ ppm 7.67 (d, *J* = 7.8 Hz, 4H), 7.44–7.40 (m, 6H), 4.51 (dd, *J* = 6.0, 12.4 Hz, 1H), 3.71 (dd, *J* = 6.7, 10.1 Hz, 1H), 3.56 (dd, *J* = 7.7, 9.6 Hz, 1H), 2.08–2.02 (m, 1H), 1.96–1.90 (m, 1H), 1.89–1.81 (m, 1H), 1.63 (br, 1H), 1.59 (ddd, *J* = 4.7, 10.1, 13.7 Hz, 1H), 1.40 (d, *J* = 6.5 Hz, 3H), 1.37–1.30 (m, 1H), 1.13 (s, 3H), 1.05 (s, 9H), 0.96 (s, 3H), 0.90 (s, 3H).

^13^C-NMR (150 MHz, CDCl_3_, 298 K): δ ppm 135.6 (d), 134.0 (s), 133.9 (s), 129.5 (d), 129.5 (d), 127.6 (d), 90.1 (s), 83.9 (s), 66.0 (t), 58.6 (d), 48.1 (t), 45.5 (s), 45.3 (s), 45.3 (s), 37.5 (t), 37.4 (t), 26.9 (q), 25.3 (t), 24.9 (q), 24.0 (q), 23.4 (q), 20.6(q), 19.2 (s).

HRMS (ESI): *m/z*: calculated for C_29_H_40_O_2_Si [M + Na^+^]: 471.2695, found: 471.2696.

Column chromatography: *n*-hexane/EtOAc (gradient 95:5–80:20).

*3-((1R,3S)-3-(((tert-butyldiphenylsilyl)oxy)methyl)-1,2,2-trimethylcyclopentyl)-1-phenylprop-2-yn-1-ol* (**8**). ^1^H-NMR (600 MHz, CDCl_3_, 298 K): δ ppm 7.66 (d, *J* = 7.5 Hz, 8H), 7.54 (d, *J* = 7.5 Hz, 4H), 7.44–7.40 (m, 4H), 7.40–7.34 (m, 12H), 7.33–7.29 (m, 2H), 5.45 (s, 2H), 3.71 (dd, *J* = 6.9, 10.0 Hz, 2H), 3.57 (dd, *J* = 7.6, 9.6 Hz, 2H), 2.10–2.04 (m, 2H), 2.03–1.97 (m, 4H), 1.90–1.83 (m, 2H), 1.67–1.61 (br, 2H), 1.38–1.31 (m, 2H), 1.19 (s, 3H), 1.18 (s, 3H), 1.04, (s, 18H), 0.97 (s, 3H), 0.98 (s, 3H), 0.93 (s, 3H), 0.92 (s, 3H).

^13^C-NMR (150 MHz, CDCl_3_, 298 K): δ ppm 141.4 (s), 141.4 (s), 135.6 (d), 134.0 (s), 133.9 (s), 129.5 (d), 129.5 (d), 128.5 (d), 128.1 (d), 127.6 (d), 126.7 (d), 93.1 (s), 81.5 (s), 65.9 (t), 64.9 (d), 48.1 (d), 45.8 (s), 45.5 (s), 37.5 (t), 37.4 (t), 26.9 (q), 25.3 (t), 24.1 (q), 24.0 (q), 23.4 (q), 20.7 (q), 20.7 (q), 19.2 (s).

HRMS (ESI): *m/z*: calculated for C_34_H_42_O_2_Si [M + Na^+^]: 533.2852, found: 533.2857.

Column chromatography: *n*-hexane/EtOAc (gradient 95:5–90:10).

*1-((1R,3S)-3-(((tert-butyldiphenylsilyl)oxy)methyl)-1,2,2-trimethylcyclopentyl)hept-6-en-1-yn-3-ol* (**9**). ^1^H-NMR (600 MHz, CDCl_3_, 298 K): δ ppm 7.68–7.65 (m, 8H), 7.44–7.36 (m, 12H), 5.87–5.80, (m, 2H), 5.05, (dd, *J* = 1.6, 17.1 Hz, 2H), 4.98 (ddd, *J* = 0.9, 0.9, 10.2 Hz, 2H), 4.38 (ddd, *J* = 2.2, 6.5, 6.5 Hz, 2H), 3.71 (dd, *J* = 6.8, 10.0 Hz, 2H), 3.56 (dd, *J* = 7.4, 10.0 Hz, 2H), 2.24–2.19 (m, 4H), 2.08–2.02 (m, 2H), 1.97–1.91 (m, 2H), 1.89–1.81 (m, 2H), 1.80–1.70 (m, 4H), 1.62–1.57 (m, 4H), 1.36–1.3 (m, 2H), 1.14 (s, 6H), 1.05 (s, 18H), 0.96 (s, 6H), 0.91 (s, 3H), 0.90 (s, 3H).

^13^C-NMR (150 MHz, CDCl_3_, 298 K): δ ppm 137.9 (d), 135.6 (d), 134.0 (s), 133.9 (s), 129.5 (d), 129.5 (d), 127.6 (d), 115.1 (t), 91.3 (s), 82.6 (s), 66.0 (t), 62.3 (d), 48.1 (d), 45.7 (s), 45.3 (s), 37.5 (t), 37.4 (t), 29.6 (t), 26.9 (q), 25.3 (t), 24.1 (q), 23.9 (q), 20.6 (q), 19.2 (s).

HRMS (ESI): *m/z*: calculated for C_32_H_44_O_2_Si [M + Na^+^]: 511.3008, found: 511.3015.

Column chromatography: *n*-hexane/EtOAc (gradient 95:5–90:10).

#### 3.5.5. Compounds **10**–**14**

Compounds **5**–**9** were each dissolved in THF (0.1 M concentration), and 1.5 equiv of TBAF solution (1.0 M in THF) was added to the mixture, which was stirred until monitorization by TLC showed that reaction was finished. Next, 15 mL of water was added to the reaction mixture, and after that it was extracted with Et_2_O (3 × 20 mL). The organic layer was collected, dried over anhydrous MgSO_4_, then filtered and concentrated by rotavap. The crude was purified by flash chromatography (silica gel) to furnish products **10**–**14**.

*1-((1R,3S)-3-(hydroxymethyl)-1,2,2-trimethylcyclopentyl)non-1-yn-3-ol* (**10**). H-NMR (500 MHz, CDCl_3_, 298 K): δ ppm 4.37 (t, *J* = 6.6 Hz, 1H), 3.74 (dd, *J* = 5.6, 10.2 Hz, 1H), 3.55 (dd, *J* = 8.0, 10.2 Hz, 1H), 2.04–1.96 (m, 2H), 1.95–1.88 (m, 1H), 1.73–1.60 (m, 4H), 1.47–1.38 (m, 3H), 1.36–1.24 (m, 7H), 1.15 (s, 3H), 0.99 (s, 3H), 0.94 (s, 3H), 0.88 (t, *J* = 6.9 Hz, 3H).

^13^C-NMR (150 MHz, CDCl_3_, 298 K): δ ppm 90.6 (s), 83.2 (s), 65.4 (t), 62.8 (d), 48.6 (d), 45.6 (s), 45.3 (s), 45.3 (s), 38.3 (t), 37.5 (t), 37.5 (t), 31.7 (t), 28.9 (t), 25.6 (t), 25.2 (t), 23.9 (q), 23.9 (q), 23.4 (q), 22.5 (t), 20.7 (q), 14.0 (q).

HRMS (ESI): *m/z*: calculated for C_18_H_32_O_2_ [M + Na^+^]: 303.2300, found: 303.2303.

Column chromatography: *n*-hexane:EtOAc (50:50).

*3-((1R,3S)-3-(hydroxymethyl)-1,2,2-trimethylcyclopentyl)prop-2-yn-1-ol* (**11**). ^1^H-NMR (500 MHz, CDCl_3_, 298 K): δ ppm 4.28 (d, *J* = 4.5 Hz, 2H), 3.74 (dd, *J* = 5.5, 10.2 Hz, 1H), 3.57 (dd, *J* = 8.2, 10.0 Hz, 1H), 2.04–1.89 (m, 3H), 1.69–1.63 (m, 1H), 1.54 (br, 1H), 1.47–1.39 (m, 1H), 1.27 (br, 1H), 1.16 (s, 3H), 0.99 (s, 3H), 0.95 (s, 3H).

^13^C-NMR (125 MHz, CDCl_3_, 298 K): δ ppm 91.6 (s), 80.2 (s), 65.3 (t), 51.5 (t), 48.5 (d), 45.6 (s), 45.3 (s), 37.5 (t), 25.5 (t), 23.9 (q), 23.3 (q), 20.7 (q).

HRMS (ESI): *m/z*: calculated for C_12_H_20_O_2_ [M + Na^+^]: 219.1361, found: 219.1369.

Column chromatography: *n*-hexane/EtOAc (40:60).

*4-((1R,3S)-3-(hydroxymethyl)-1,2,2-trimethylcyclopentyl)but-3-yn-2-ol* (**12**). ^1^H-NMR (600 MHz, CDCl_3_, 298 K): δ ppm 4.54 (dd, *J* = 6.5, 13.1 Hz, 1H), 3.74 (dd, *J* = 5.7, 10.4 Hz, 1H), 3.56 (dd, *J* = 8.3, 10.1 Hz, 1H), 2.03–1.89 (m, 3H), 1.68–1.62 (m, 1H), 1.43 (d, *J* = 6.5 Hz, 4H), 1.15 (s, 3H), 0.98 (s, 3H), 0.94 (s, 3H).

^13^C-NMR (150 MHz, CDCl_3_, 298 K): δ ppm 89.9 (s), 84.1 (s), 65.4 (t), 58.6 (d), 48.6 (d), 45.5 (s), 45.4 (s), 45.3 (s), 37.5 (t), 37.5 (t), 25.6 (t), 24.9 (q), 23.9 (q), 23.4 (q), 20.6 (q).

HRMS (ESI): *m/z*: calculated for C_13_H_22_O_2_ [M + Na^+^]: 233.1517, found: 233.1513.

Column chromatography: *n*-hexane/EtOAc (50:50).

*3-((1R,3S)-3-(hydroxymethyl)-1,2,2-trimethylcyclopentyl)-1-phenylprop-2-yn-1-ol* (**13**). ^1^H-NMR (600 MHz, CDCl_3_, 298 K): δ ppm 7.55 (d, *J* = 7.4 Hz, 4H), 7.38 (t, *J* = 7.5 Hz, 4H), 7.32 (t, *J* = 7.3 Hz, 2H), 5.48 (s, 2H), 3.73 (dd, *J* = 5.6, 10.3 Hz, 2H), 3.54 (dd, *J* = 8.1, 10.1 Hz, 1H), 2.12 (br, 2H), 2.09–1.91 (m, 6H), 1.73–1.67 (m, 2H), 1.58 (br, 2H), 1.48–1.41 (m, 2H), 1.21 (s, 3H), 1.21 (s, 3H), 1.01 (s, 3H), 1.01 (s, 3H), 0.97 (s, 3H), 0.96 (s, 3H).

^13^C-NMR R (150 MHz, CDCl_3_, 298 K): δ ppm 141.4 (s), 128.5 (d), 128.2 (d), 126.7 (d), 92.8 (s), 81.8 (s), 65.3 (t), 64.9 (d), 48.6 (d), 45.8 (s), 45.5 (s), 37.5 (t), 37.5 (t), 25.6 (t), 23.9 (q), 23.9 (q), 23.4 (q), 20.8 (q).

HRMS (ESI): *m/z*: calculated for C_18_H_24_O_2_ [M + Na^+^]: 295.1674, found: 295.1675.

Column chromatography: DCM/EtOAc (gradient 90:10–50:50).

*1-((1R,3S)-3-(hydroxymethyl)-1,2,2-trimethylcyclopentyl)hept-6-en-1-yn-3-ol* (**14**). ^1^H-NMR (500 MHz, CDCl_3_, 298 K): δ ppm 5.89–5.79, (m, 1H), 5.07, (dd, *J* = 1.7, 17.1 Hz, 1H), 5.00–4.96 (m, 1H), 4.40 (t, *J* = 6.5 Hz, 1H), 3.73 (dd, *J* = 5.6, 10.2 Hz, 1H), 3.56 (dd, *J* = 8.0, 10.2 Hz, 1H), 2.28–2.16 (m, 2H), 2.05–1.88 (m, 3H), 1.84–1.71 (m, 3H), 1.69–1.62 (m, 1H), 1.46–1.38 (m, 1H), 1.16 (s, 3H), 0.99 (s, 3H), 0.94 (s, 3H).

^13^C-NMR (150 MHz, CDCl_3_, 298 K): δ ppm 137.9 (d), 115.1 (t), 91.0 (s), 82.9 (s), 65.3 (t), 62.2 (d), 48.5 (d), 45.6 (s), 45.3 (s), 45.3 (s), 37.5 (t), 37.5 (t), 37.3 (t), 29.6 (t), 25.6 (t), 23.9 (q), 23.4 (q), 20.7 (q).

HRMS (ESI): *m/z*: calculated for C_16_H_26_O_2_ [M + Na^+^]: 273.1831, found: 273.1834.

Column chromatography: *n*-hexane/EtOAc (70:30).

#### 3.5.6. Compounds **1**, **20**–**23**

General procedure: To a solution of the corresponding diol (**10**–**14**) in DCM (0.1 M) under inert atmosphere (Ar), we added dicobalt octacarbonyl complex (1.2 equiv), and the mixture was stirred until the reaction was finished, which was monitored by TLC. Afterwards, the solvent was evaporated in vacuo, and the crude solid was adsorbed in silica gel and then separated by flash chromatography. The resulting compounds were again dissolved in DCM (0.05 M) under inert atmosphere (Ar), and the solution was cooled to −20 °C before dropwise addition of 1.3 equiv of BF_3_·Et_2_O. The resulting mixture was placed in a fridge to maintain the temperature at −20 °C during 36 h. Next, the reaction mixture was poured in a vigorously stirred solution of saturated NaHCO_3_ (30 mL). Then, the organic layer was collected, and the aqueous layer was extracted with DCM (3 × 20 mL). The organic extracts were dried over anhydrous MgSO_4_, filtered, and concentrated under reduced pressure. The alkyne–Co_2_(CO)_6_ complexes from the crude were isolated by flash chromatography (silica gel). Finally, 6 equiv of 4-methylmorpholine *N*-oxide (NMO) were added to a DCM solution containing the resulting complexes under inert atmosphere (Ar), and the mixture was stirred for 36 h. After filtration of the NMO residues, the solvent was removed by rotavap and the crude was purified by flash chromatography (silica gel) obtaining compounds **1** and **21**–**23**. 

*(1S,4R,7R)-4-hexyl-7,10,10-trimethyl-3-oxabicyclo[5.2.1]dec-5-yne* (**1**). ^1^H-NMR (500 MHz, CDCl_3_, 298 K): δ ppm 4.24 (dd, *J* = 5.9, 6.9 Hz, 1H), 3.85 (dd, *J* = 8.7, 14.0 Hz, 1H), 3.54 (d, *J* = 14.0 Hz, 1H), 2.26–2.16 (m, 1H), 1.88–1.71 (m, 4H), 1.65–1.52 (m, 2H), 1.41–1.22 (m, 8H), 1.15 (s, 3H), 0.97 (s, 3H), 0.87 (t, 6.9 Hz, 3H), 0.86 (s, 3H).

^13^C-NMR (150 MHz, CDCl_3_, 298 K): δ ppm 110.5 (s), 93.1 (s), 73.6 (d), 67.7 (t), 54.3 (s), 50.1 (d), 47.4 (s), 38.4 (t), 34.2 (t), 31.7 (t), 29.1 (t), 26.0 (q), 25.3 (t), 22.5 (t), 20.6 (q), 18.8 (t), 15.4 (q), 14.1 (q).

HRMS (ESI): *m/z*: calculated for C_18_H_30_O [M + H^+^]: 263.2375, found: 263.2377.

Column chromatography: *n*-hexane/EtOAc (gradient 85:15–70:30).

*(1S,7R)-4,7,10,10-tetramethyl-3-oxabicyclo[5.2.1]dec-5-yne* (**21**). ^1^H-NMR (500 MHz, CDCl_3_, 298 K): δ ppm 4.33 (dd, *J* = 6.6, 13.2 Hz, 2H), 3.83 (dd, *J* = 8.6, 14.0 Hz, 1H), 3.55 (d, *J* = 14.0 Hz, 1H), 2.26–2.18 (m, 1H), 1.88–1.71 (m, 4H), 1.30 (d, *J* = 6.6 Hz, 1H), 1.14 (s, 3H), 0.97 (s, 3H), 0.86 (s, 3H).

^13^C-NMR (150 MHz, CDCl_3_, 298 K): δ ppm 109.6 (s), 93.9 (s), 68.9 (d), 67.8 (t), 54.4 (s), 50.3 (d), 47.4 (s), 38.5 (t), 26.1 (q), 20.6 (q), 20.6 (q), 18.9 (t), 15.4 (q).

HRMS (ESI): *m/z*: calculated for C_13_H_20_O [M + Na^+^]: 215.1412, found: 215.1414.

Column chromatography: *n*-hexane/DCM (gradient 90:10–80:20).

*(1S,4R,7R)-7,10,10-trimethyl-4-phenyl-3-oxabicyclo[5.2.1]dec-5-yne* (**22**). ^1^H-NMR (500 MHz, CDCl_3_, 298 K): δ ppm 7.46–7.46 (m, 2H), 7.37–7.28 (m, 3H), 5.30 (s, 1H), 3.99 (dd, *J* = 8.7, 13.9 Hz, 1H), 3.77 (d, *J* = 13.9 Hz, 1H), 2.35–2.28 (m, 1H), 1.98–1.91 (m, 1H), 1.88–1.78 (m, 1H), 1.24 (s, 3H), 1.03 (s, 3H), 0.90 (s, 3H).

^13^C-NMR (150 MHz, CDCl_3_, 298 K): δ ppm 138.1 (s), 128.4 (d), 128.4 (d), 127.5 (d), 112.4 (s), 91.3 (s), 75.5 (d), 68.2 (t), 54.5 (s), 50.2 (d), 47.5 (s), 38.5 (t), 26.0 (q), 20.7 (q), 19.0 (t), 15.3 (q).

HRMS (ESI): *m/z*: calculated for C_18_H_22_O [M + Na^+^]: 277.1568, found: 277.1570.

Column chromatography: *n*-hexane/DCM (gradient 85:15–70:30).

*(1S,4R,7R)-4-(but-3-en-1-yl)-7,10,10-trimethyl-3-oxabicyclo[5.2.1]dec-5-yne (**23**).*^1^H-NMR (500 MHz, CDCl_3_ 298 K): δ ppm 5.82–5.74 (m, 1H), 5.01 (dd, *J* = 1.7, 17.2 Hz, 1H), 4.93 (d, *J* = 10.2 Hz, 1H), 4.25 (t, *J* = 6.2 Hz, 1H), 3.81 (dd, *J* = 8.7, 14.0 Hz, 1H), 3.51 (d, *J* = 14.0 Hz, 1H), 2.20–2.07 (m, 2H), 1.86–1.59 (m, 3H), 1.12 (s, 3H), 0.95 (s, 3H), 0.83 (s, 3H).

^13^C-NMR (150 MHz, CDCl_3_, 298 K): δ ppm 137.8 (d), 114.8 (t), 111.0 (s), 92.6 (s), 72.9 (d), 67.6 (t), 54.3 (s), 50.1 (d), 47.3 (s), 38.4 (t), 33.2 (t), 29.4 (t), 26.0 (q), 20.6 (q), 18.8 (t), 15.3 (q).

HRMS (ESI): *m/z*: calculated for C_16_H_24_O [M + Na^+^]: 255.1725, found: 255.1720.

Column chromatography: *n*-hexane/EtOAc (gradient 95:5–70:30).

## 4. Conclusions

In conclusion, a series of strained 1-oxa-3-cyclooctynes were synthesized from commercially available (1*R*,3*S*)-camphoric acid. The key cyclization step is an intramolecular Nicholas reaction. The synthetic route involves the preparation of propargylic alcohols as key precursors. Firstly, a terminal alkyne was installed in the core structure through a Corey–Fuchs reaction. Subsequent generation of the corresponding alkanide anion and reaction with different aldehydes provided in good yield the desired propargylic alcohols. Complexation of the triple bond with Co_2_(CO)_8_ and treatment with a Lewis acid (BF_3_·Et_2_O) induced the intramolecular Nicholas reaction in which a free hydroxyl group acts as nucleophile. Finally, oxidative deprotection of the triple bonds with NMO afforded the desired cyclooctynes in good yields (31–64% overall yield from the propargylic alcohols). Importantly, large-sized R substituents (e.g., R = *n*-hexyl, Ph, 3-butenyl) at the chiral center connected to the O atom were oriented during the cyclization step in such a way that steric interactions were minimized, affording the corresponding cyclooctynes exclusively with *(R)* configuration. The results indicate that the preorganization of the system and steric factors are key aspects in the outcome of the cyclization. Preliminary experiments also showed that these cyclooctynes are reactive in the presence of azides, affording the corresponding substituted triazoles.

Current efforts in our laboratories are focused on the development of an analogue route for the synthesis of aza-cyclooctynes and a detailed evaluation of these compounds as building blocks for sequential catalyst-free click reactions. 

## Figures and Tables

**Figure 1:**
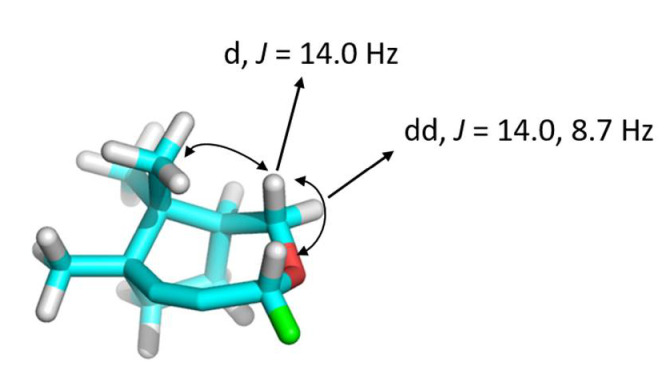
3D structural model of cyclooctyne (*R*)-**1**. Black arrows indicate nuclear Overhauser effects (NOEs), and the alkyl group is represented in green color.

## Data Availability

Data is contained within the article and supplementary material.

## Supplementary Materials

NMR spectra (Figures S1–S47) are available online.

## Sample Availability

Samples of the compounds are available from the authors.
