# Supplementary material for: Intramolecular Nicholas Reaction Enables the Stereoselective Synthesis of Strained Cyclooctynes
Source: Molecules. 2021 Mar 15;26(6):1629. doi: 10.3390/molecules26061629 (PMC7999112; doi:10.3390/molecules26061629)
Supplement: Supplementary file 1 [file molecules-26-01629-s001.zip › molecules-1148643-supplementary.pdf]

Supporting Information

Intramolecular Nicholas Reaction Enables the Synthesis of Strained Cyclooctynes

Diego Manuel Monzón, Juan Manuel Betancort, Tomás Martín, Miguel Ángel Ramírez, Víctor S. Martín* and David Díaz Díaz*

NMR spectra of compounds

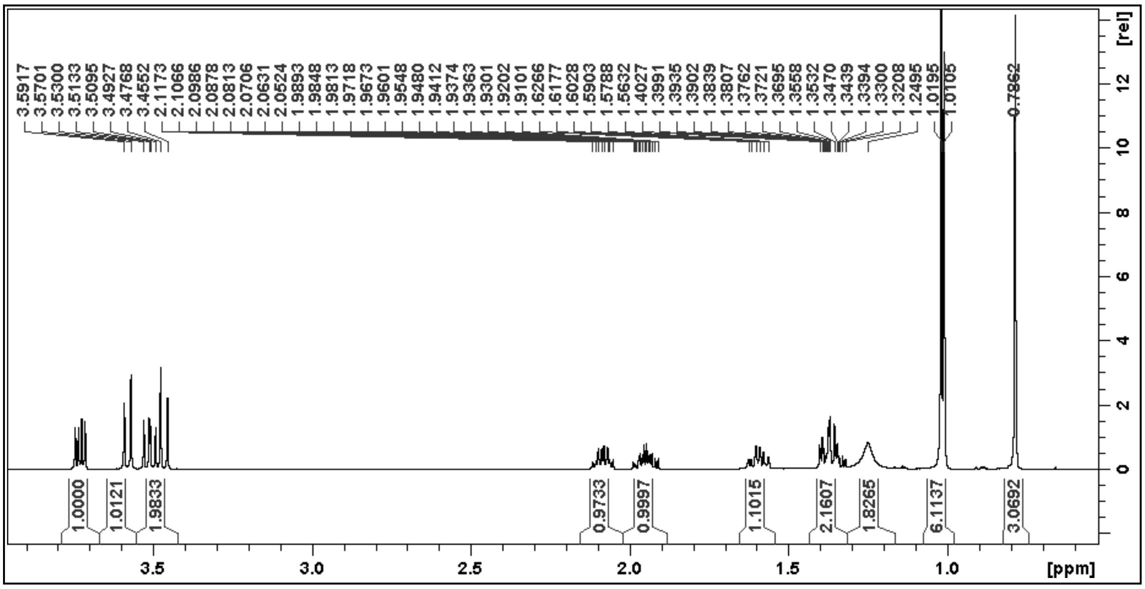

Figure S1. ^{1}H NMR spectrum of compound **2**

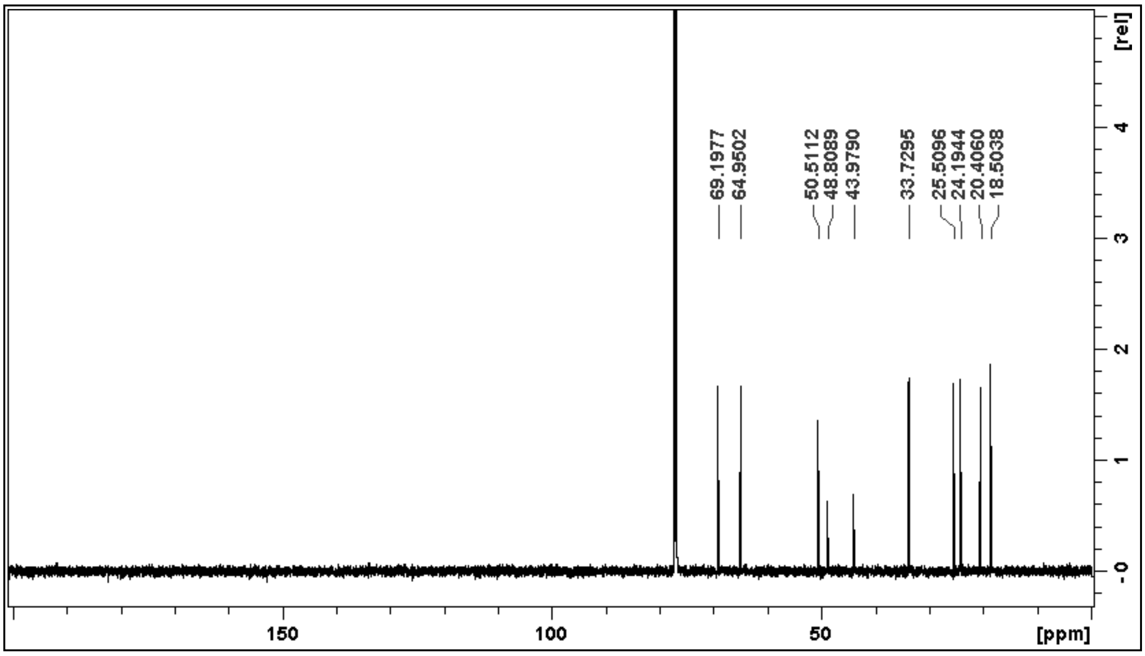

Figure S2. ^{13}C NMR spectrum of compound **2**

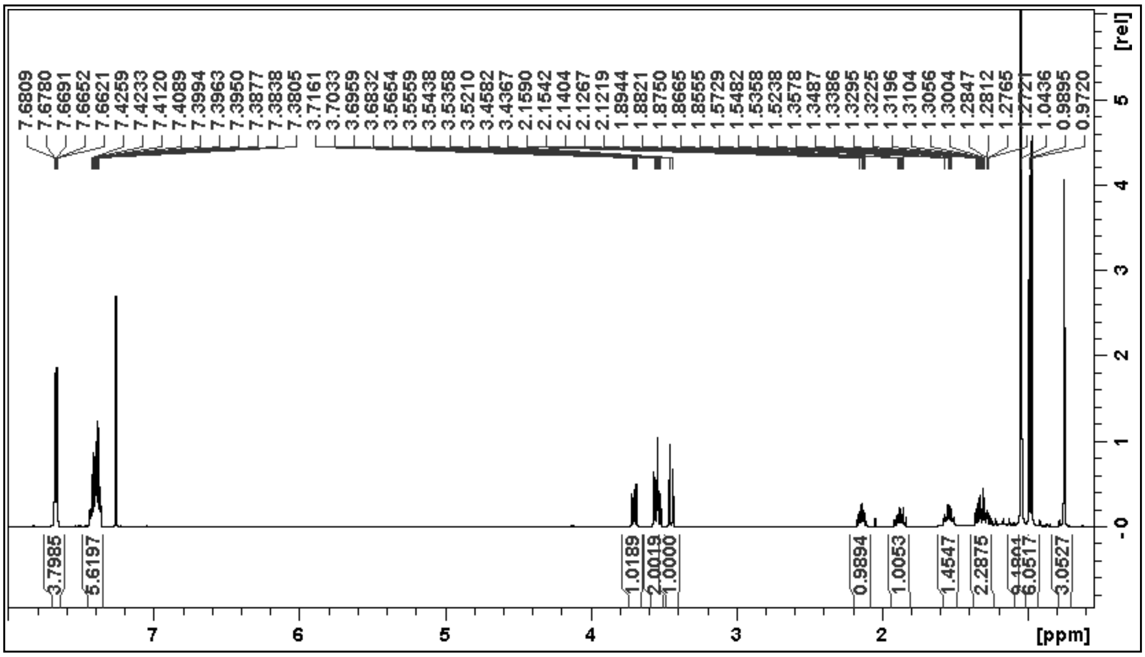

Figure S3. ^{1}H NMR spectrum of compound **3**

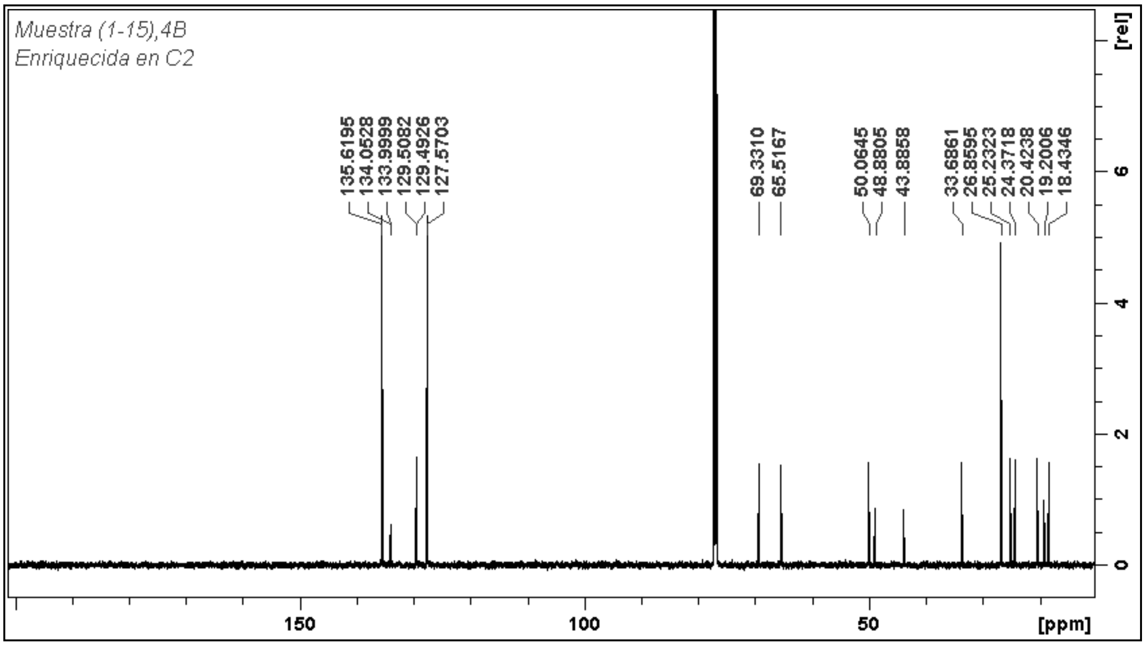

Figure S4. ^{13}C NMR spectrum of compound **3**

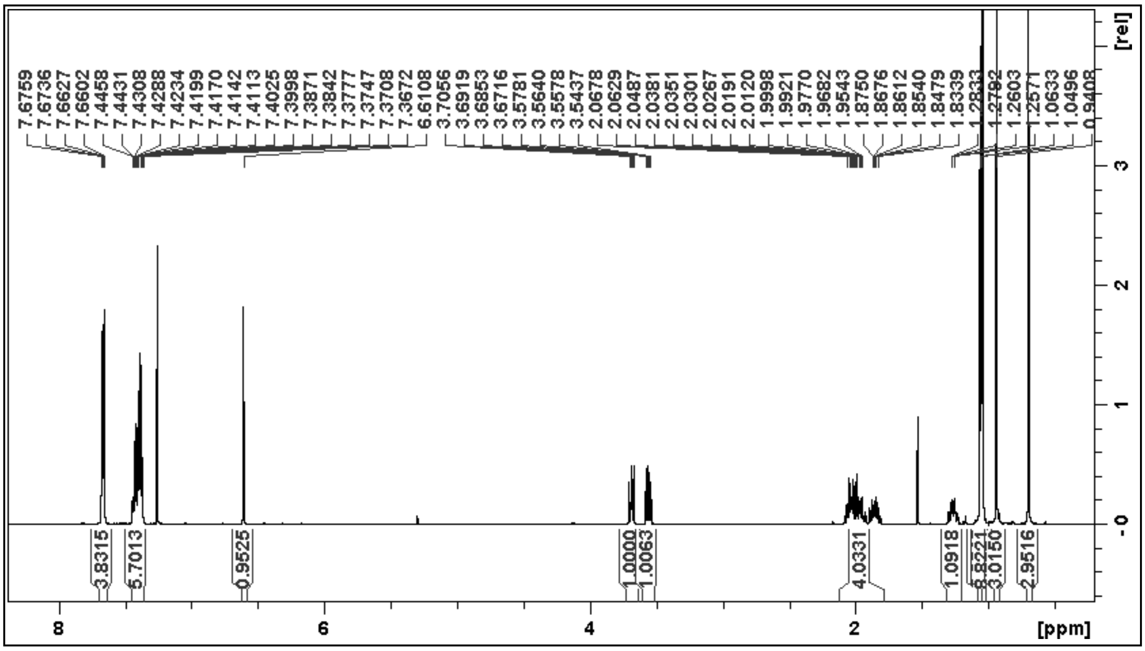

Figure S5. ^{1}H NMR spectrum of compound **4**

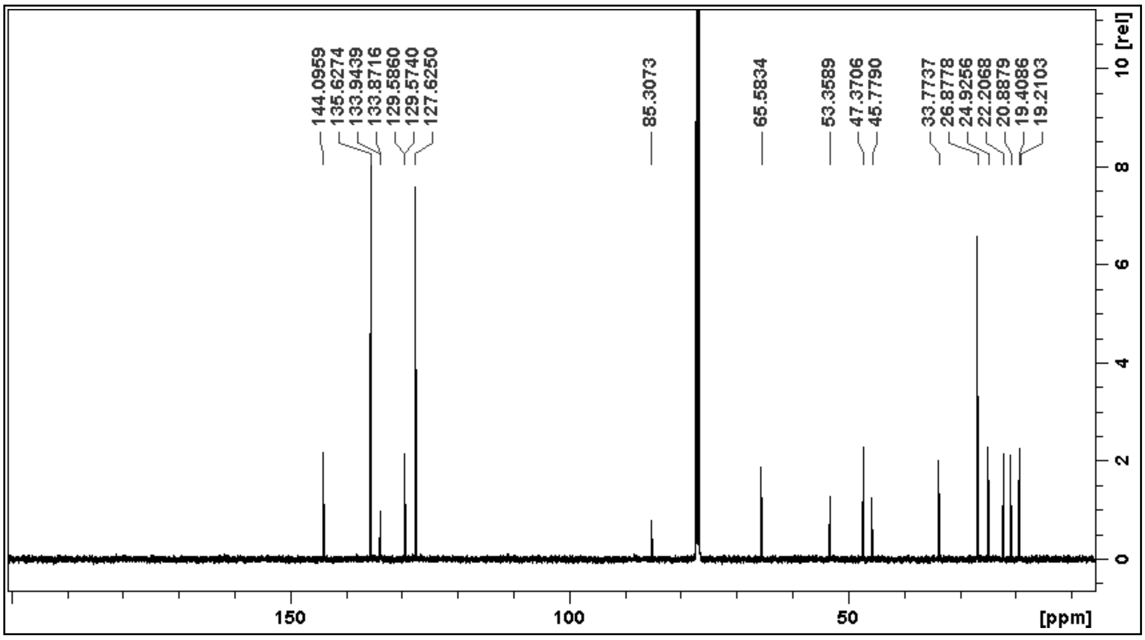

Figure S6. ^{13}C NMR spectrum of compound **4**

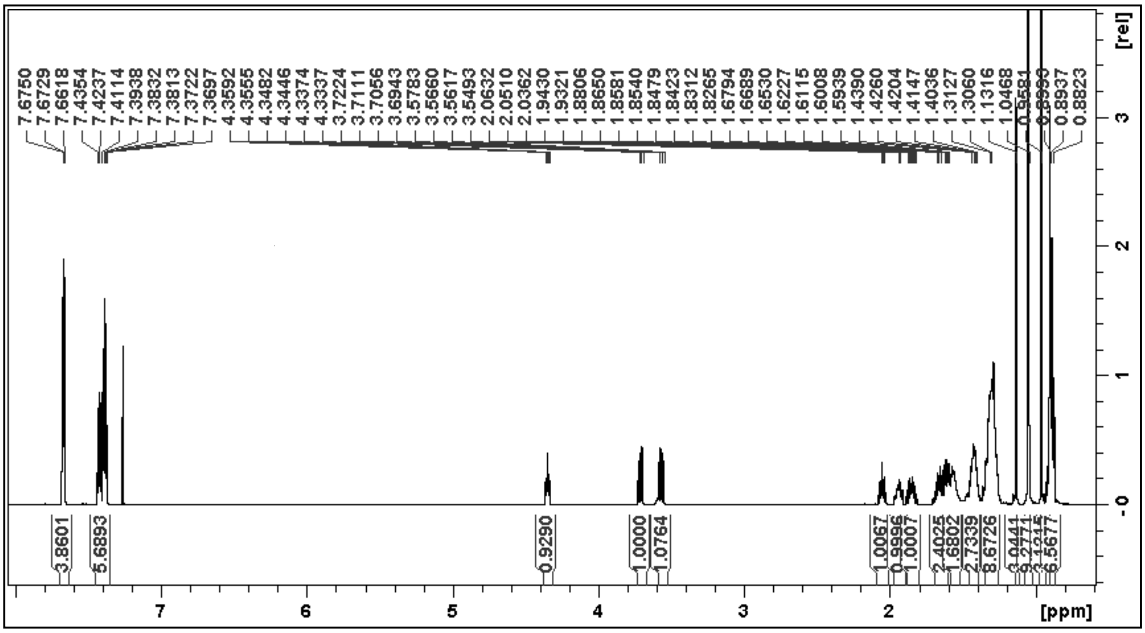

Figure S7. ^{1}H NMR spectrum of compound **5**

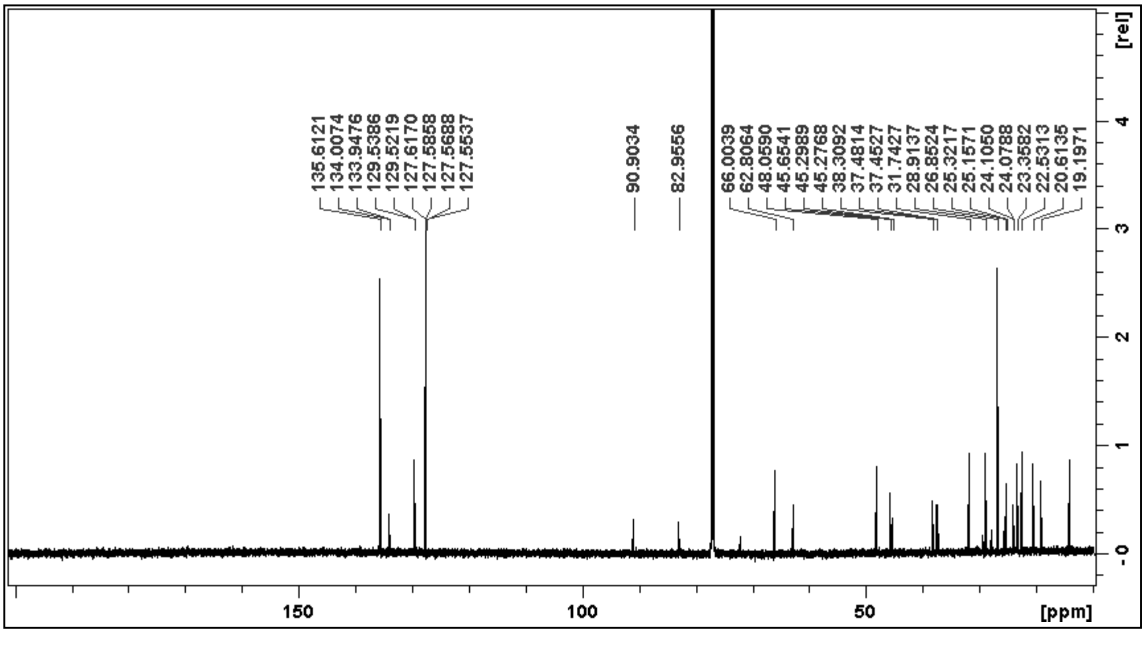

Figure S8. ^{13}C NMR spectrum of compound **5**

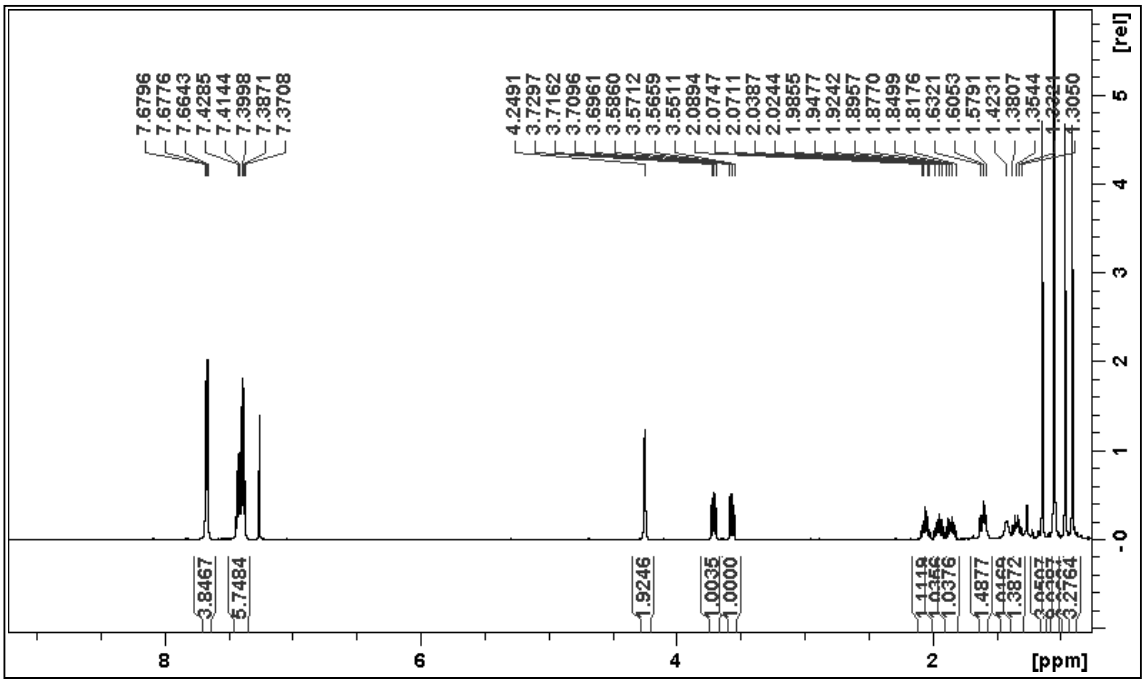

Figure S9. ^{1}H NMR spectrum of compound **6**

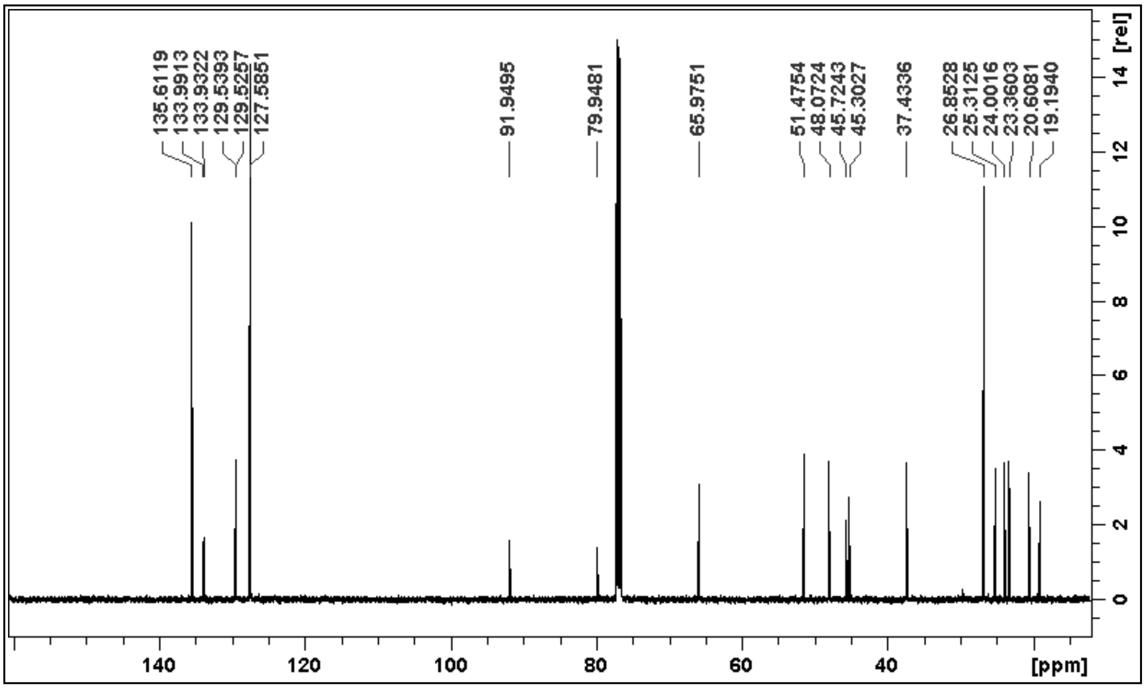

Figure S10. ^{13}C NMR spectrum of compound **6**

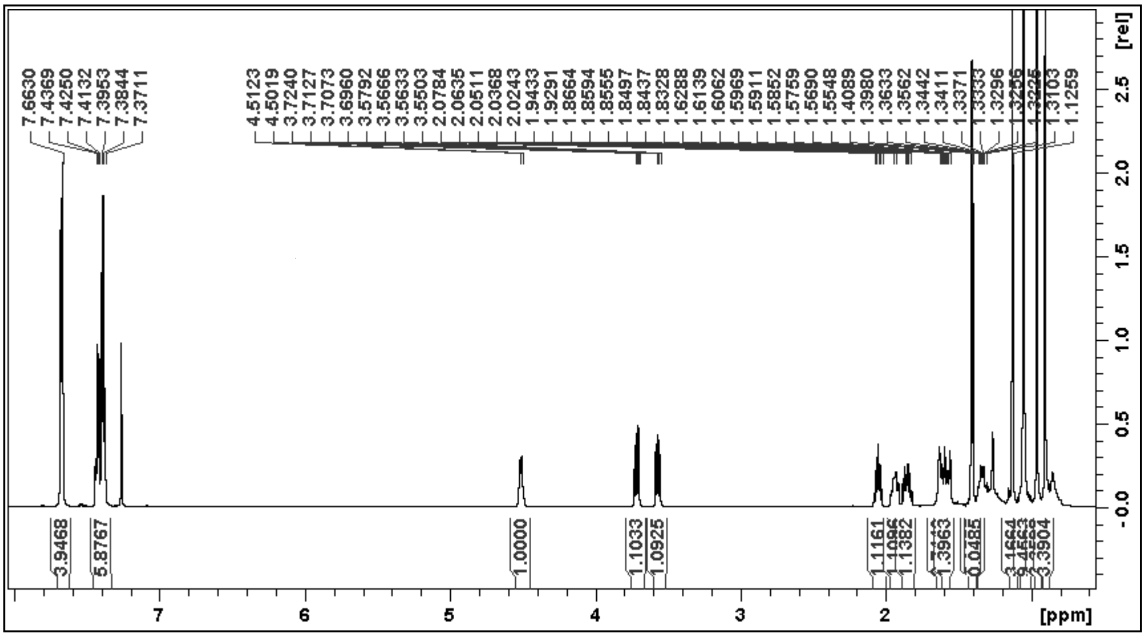

Figure S11. ^{1}H NMR spectrum of compound **7**

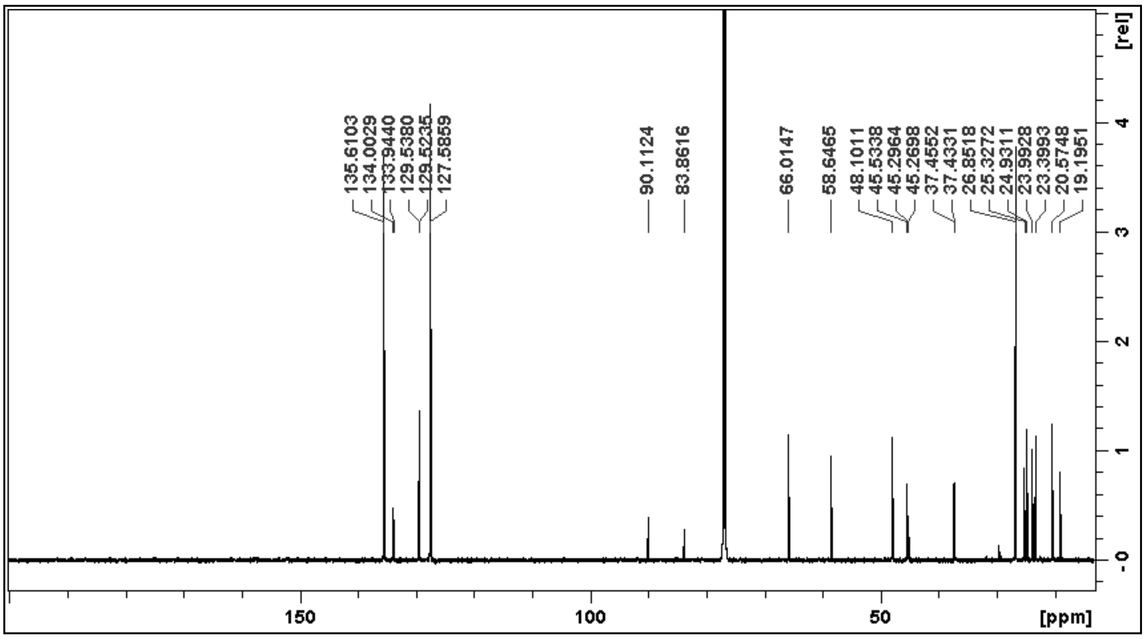

Figure S12. ^{13}C NMR spectrum of compound **7**

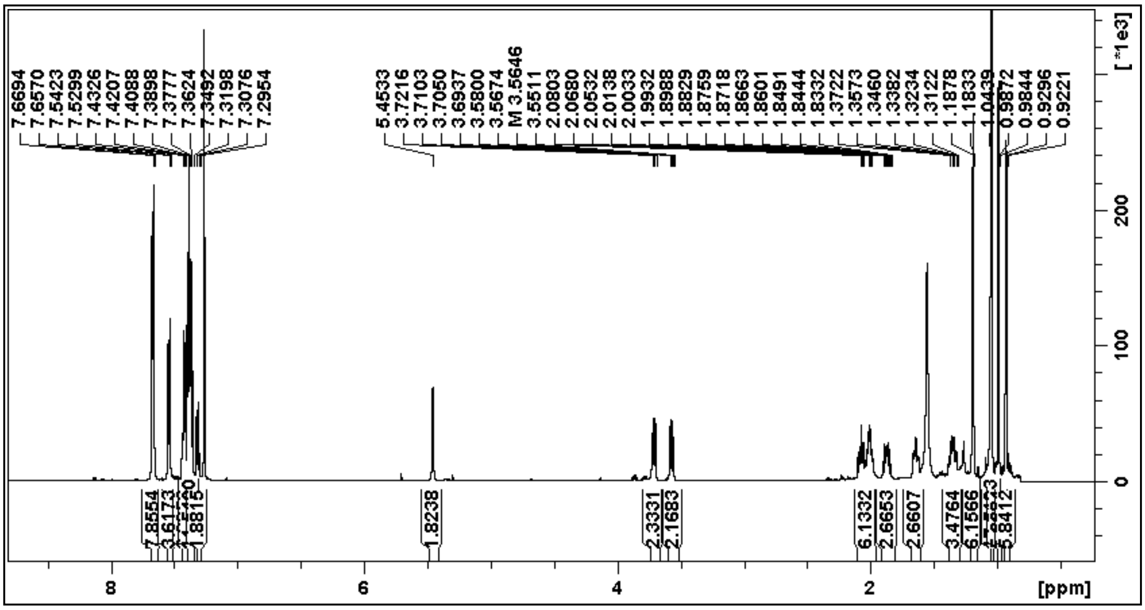

Figure S13. ^{1}H NMR spectrum of compound **8**

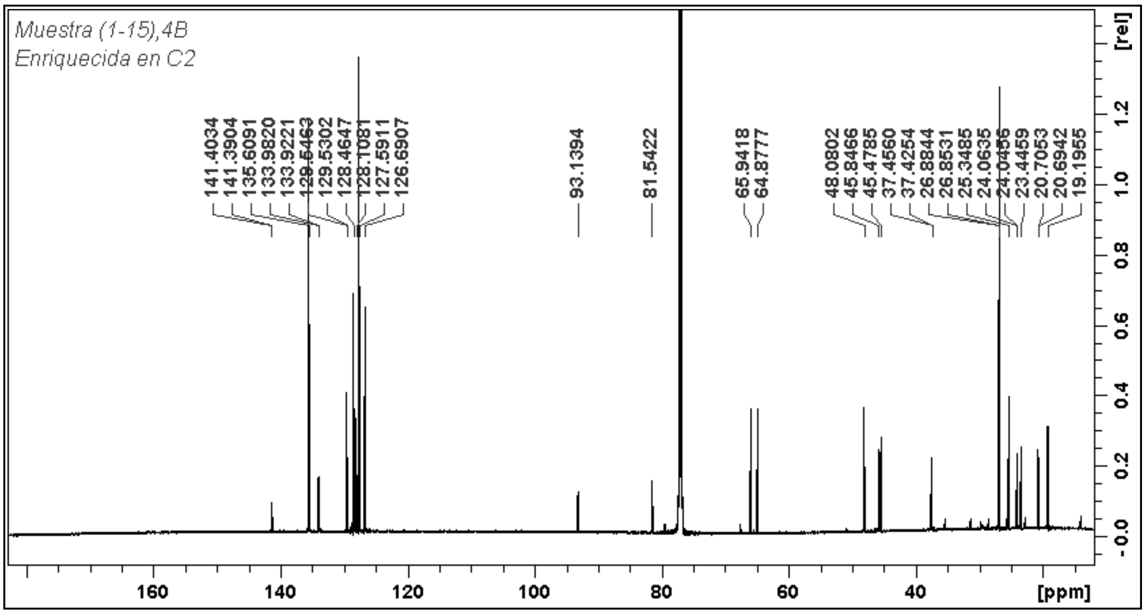

Figure S14. ^{13}C NMR spectrum of compound **8**

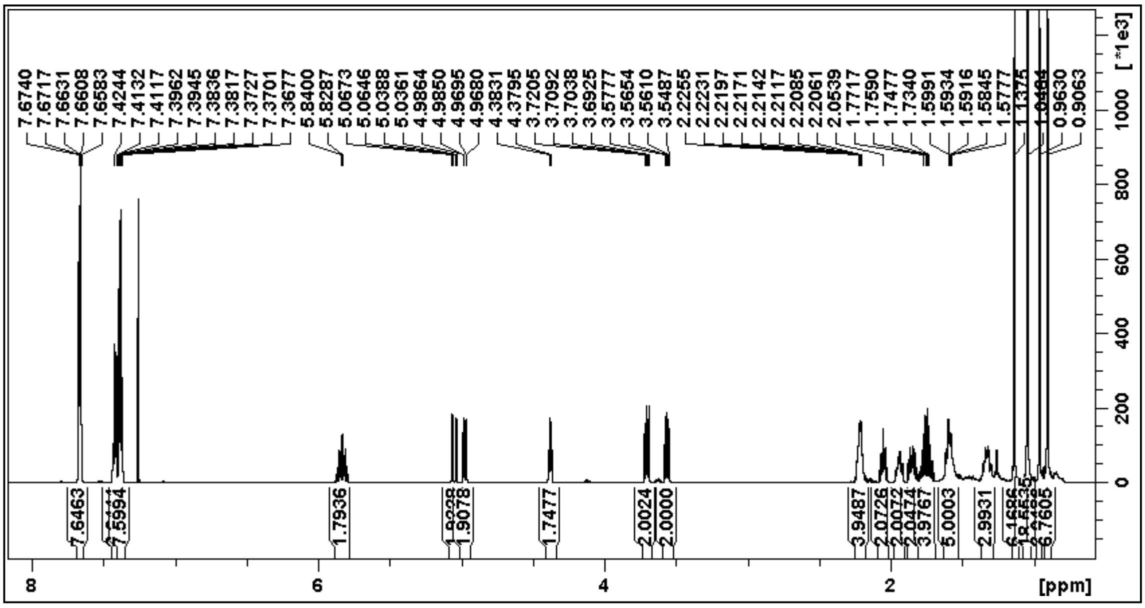

Figure S15. ^{1}H NMR spectrum of compound **9**

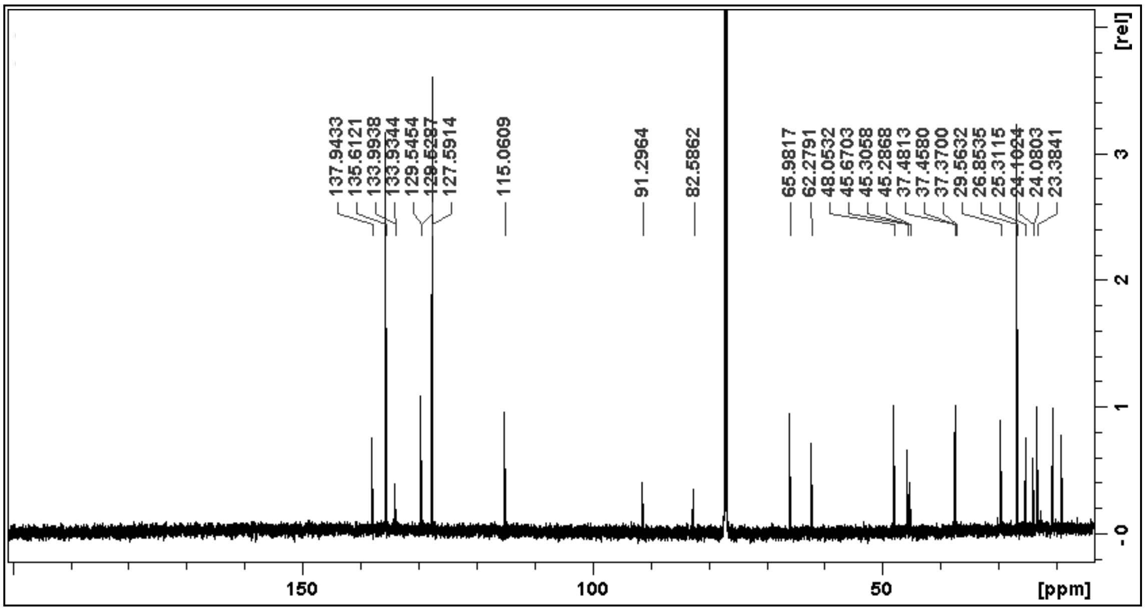

Figure S16. ^{13}C NMR spectrum of compound **9**

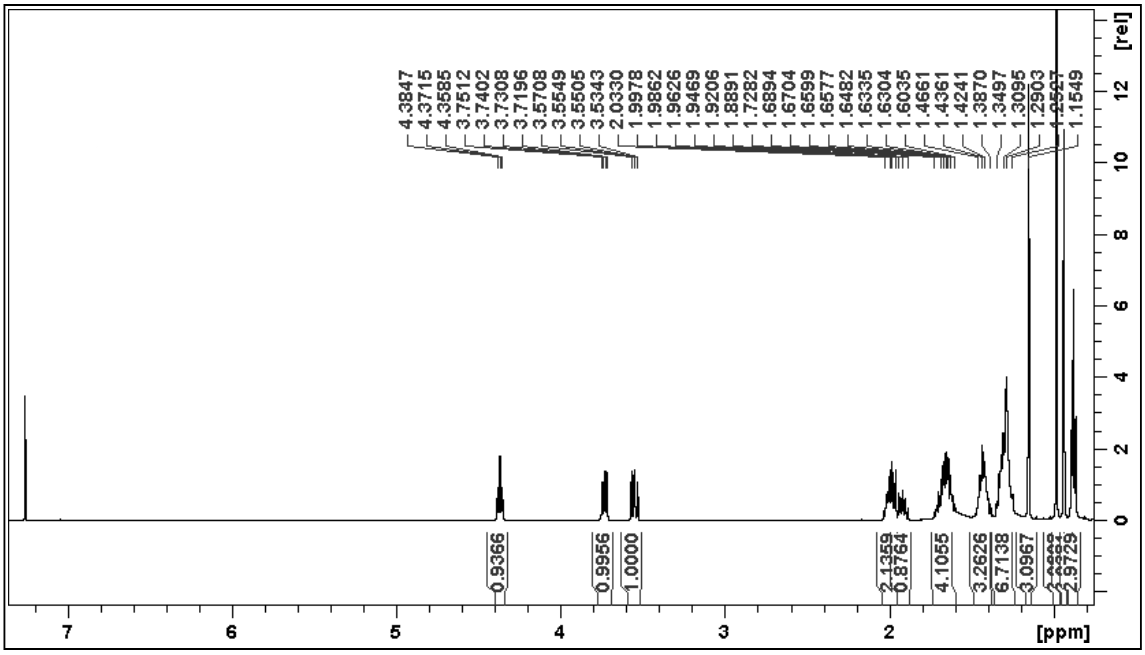

Figure S17. ^{1}H NMR spectrum of compound **10**

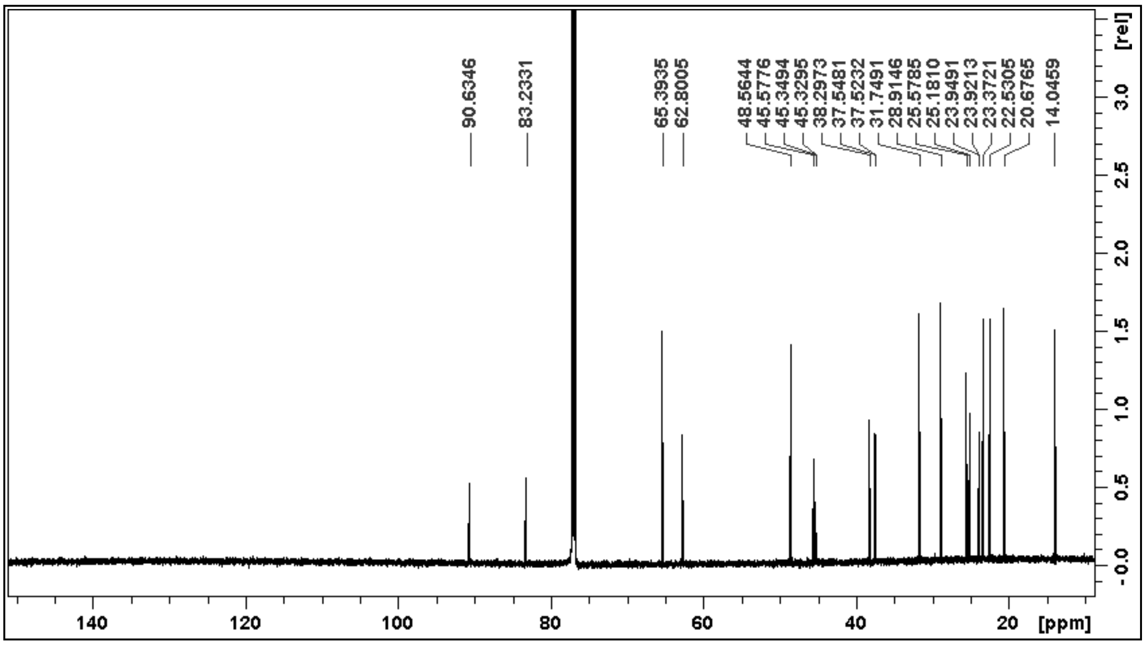

Figure S18. ^{13}C NMR spectrum of compound **10**

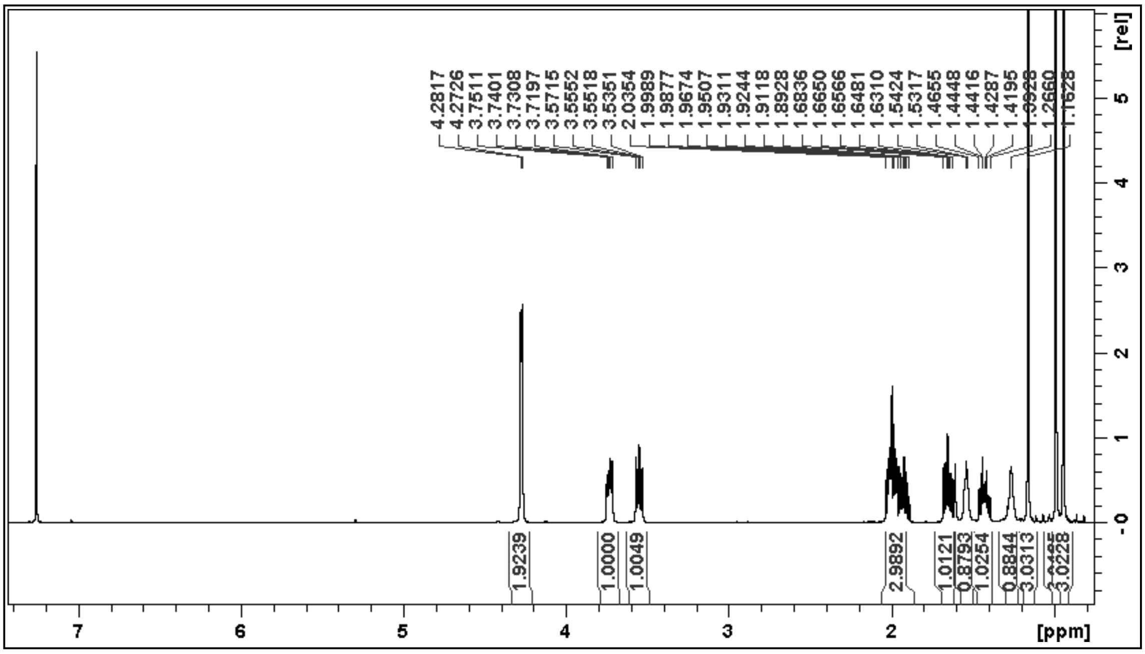

Figure S19. ^{1}H NMR spectrum of compound **11**

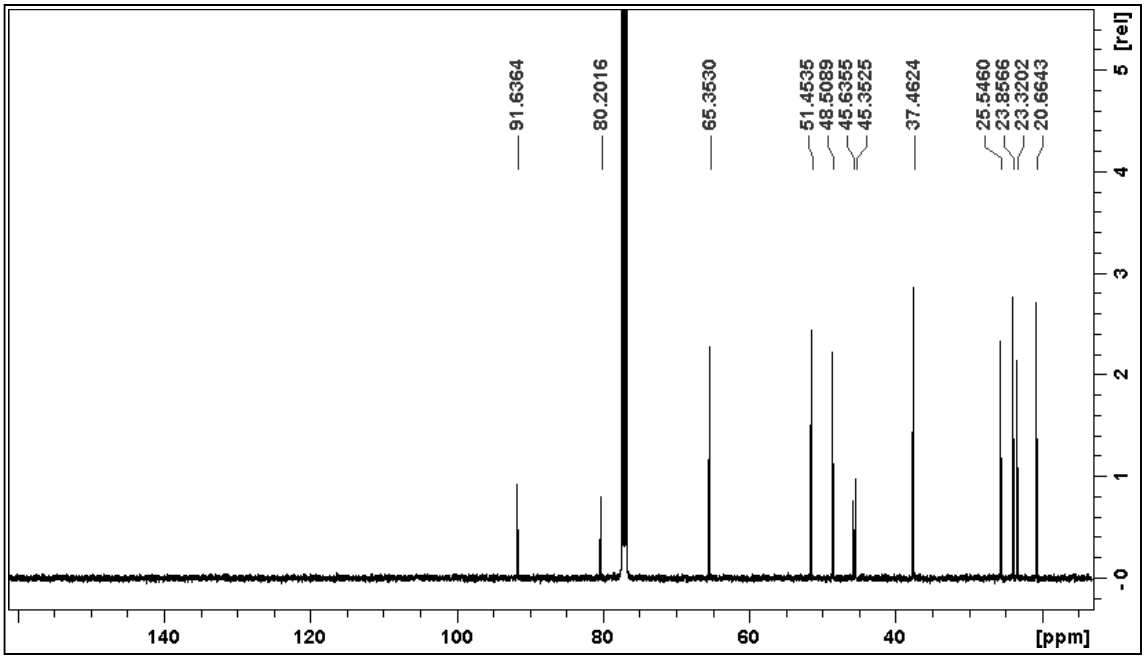

Figure S20. ^{13}C NMR spectrum of compound **11**

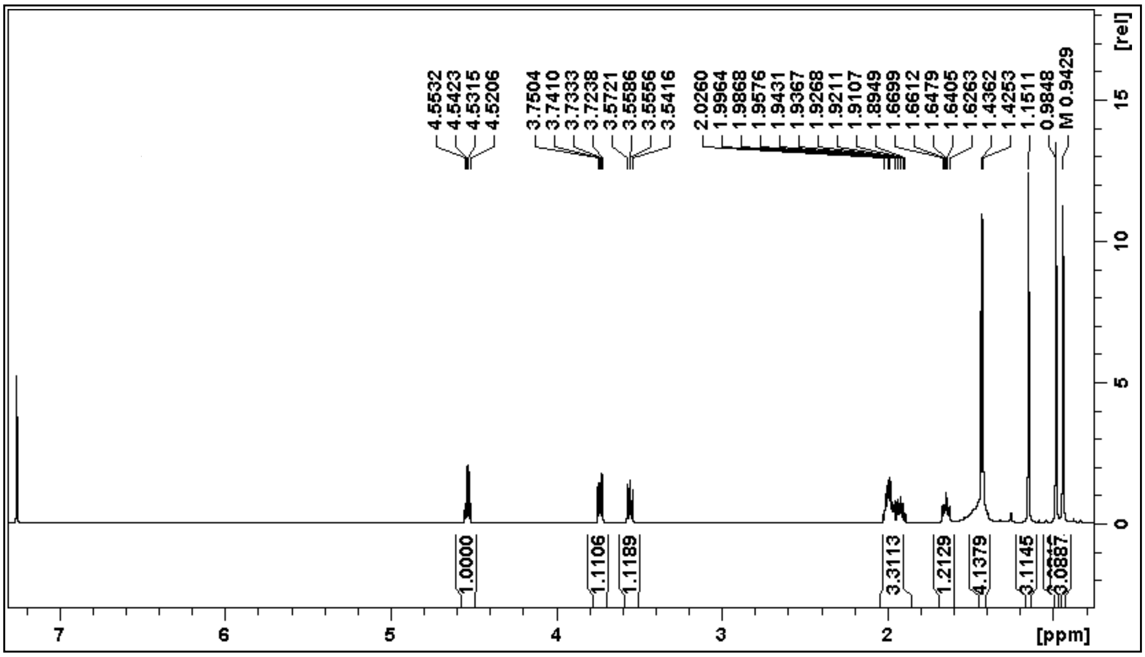

Figure S21. ^{1}H NMR spectrum of compound **12**

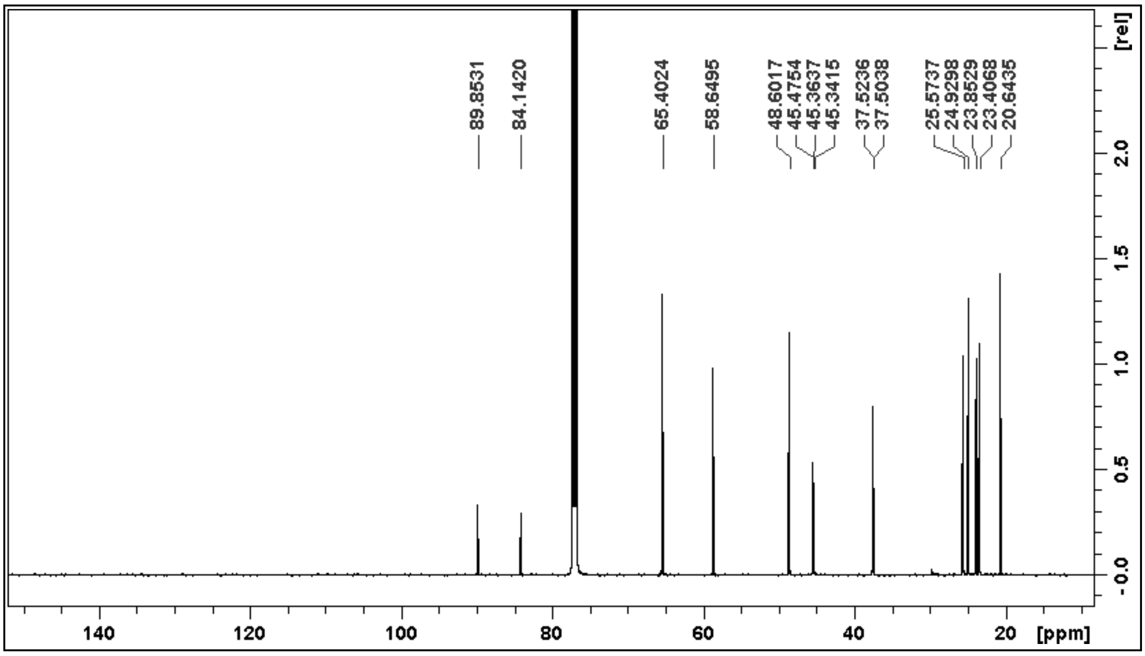

Figure S22. ^{13}C NMR spectrum of compound **12**

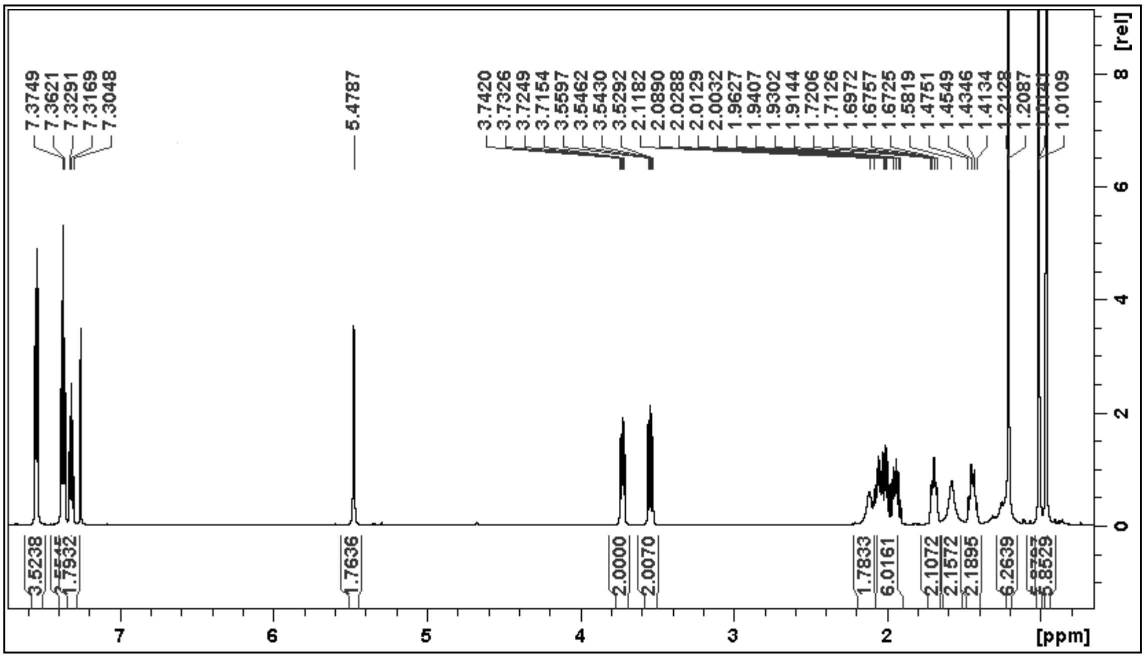

Figure S23. ^{1}H NMR spectrum of compound **13**

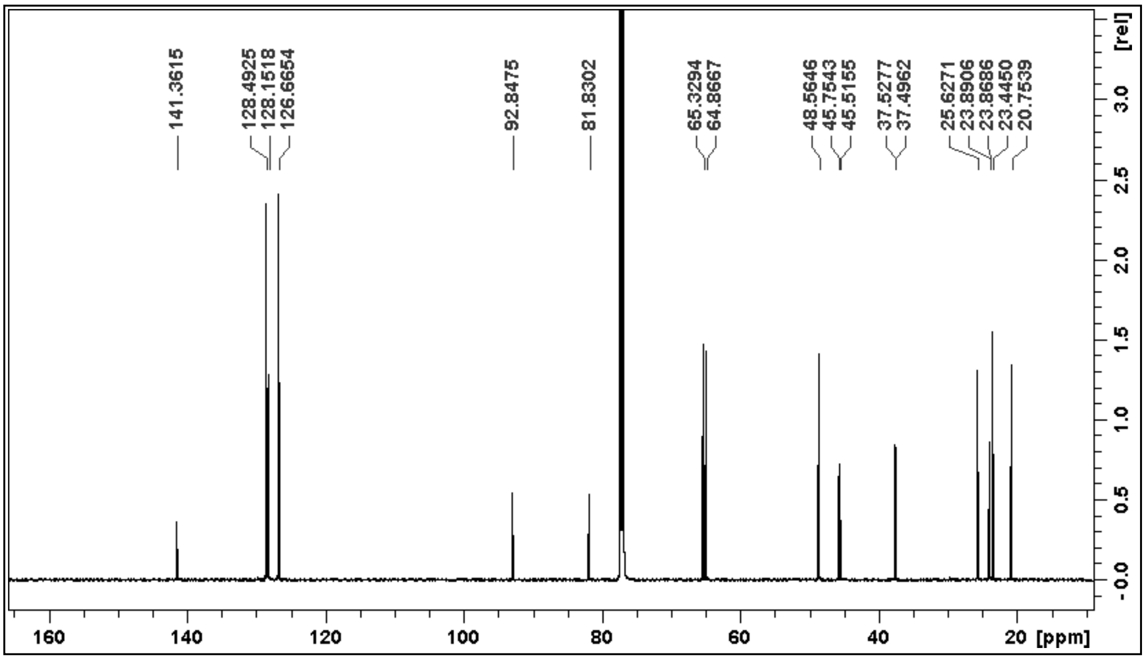

Figure S24. ^{13}C NMR spectrum of compound **13**

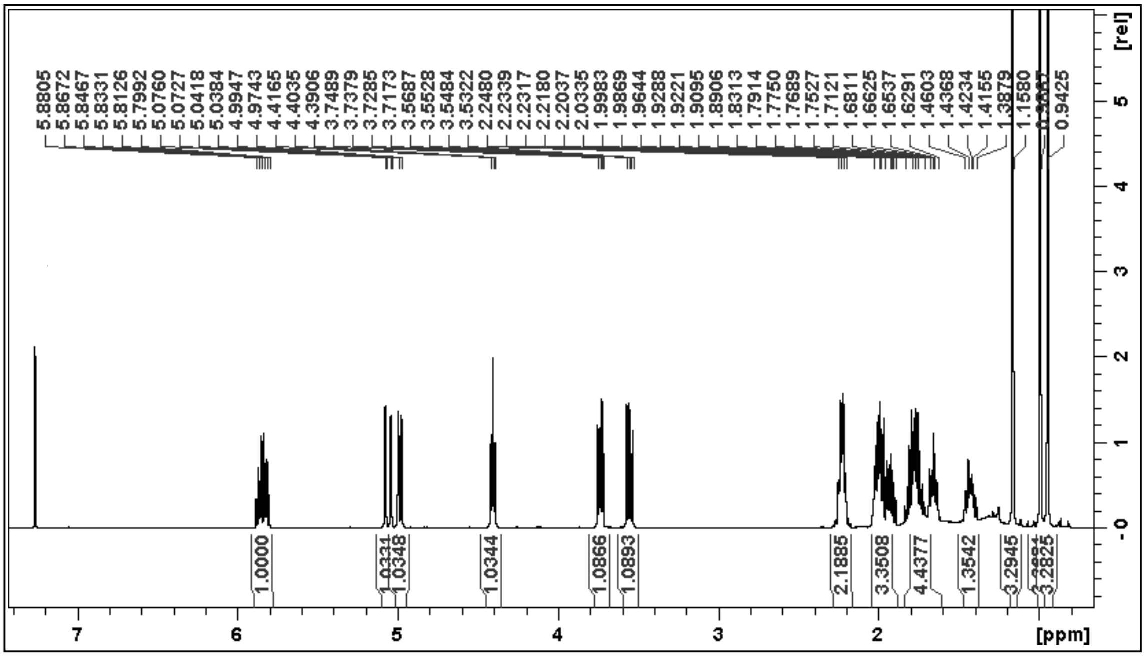

Figure S25. ^{1}H NMR spectrum of compound **14**

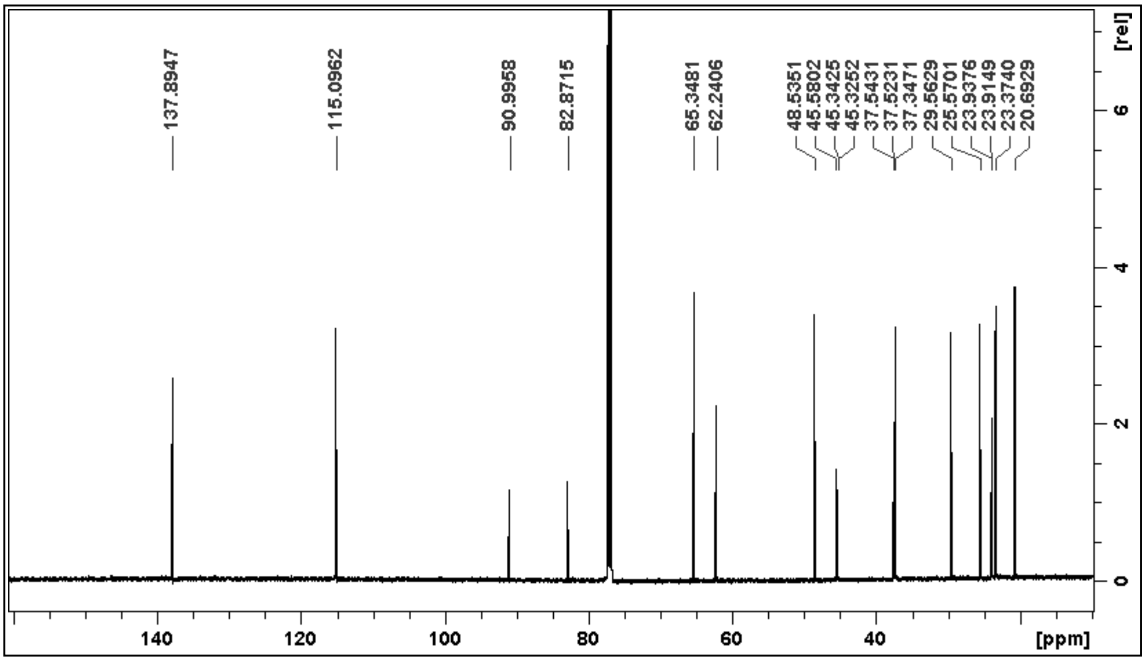

Figure S26. ^{13}C NMR spectrum of compound **14**

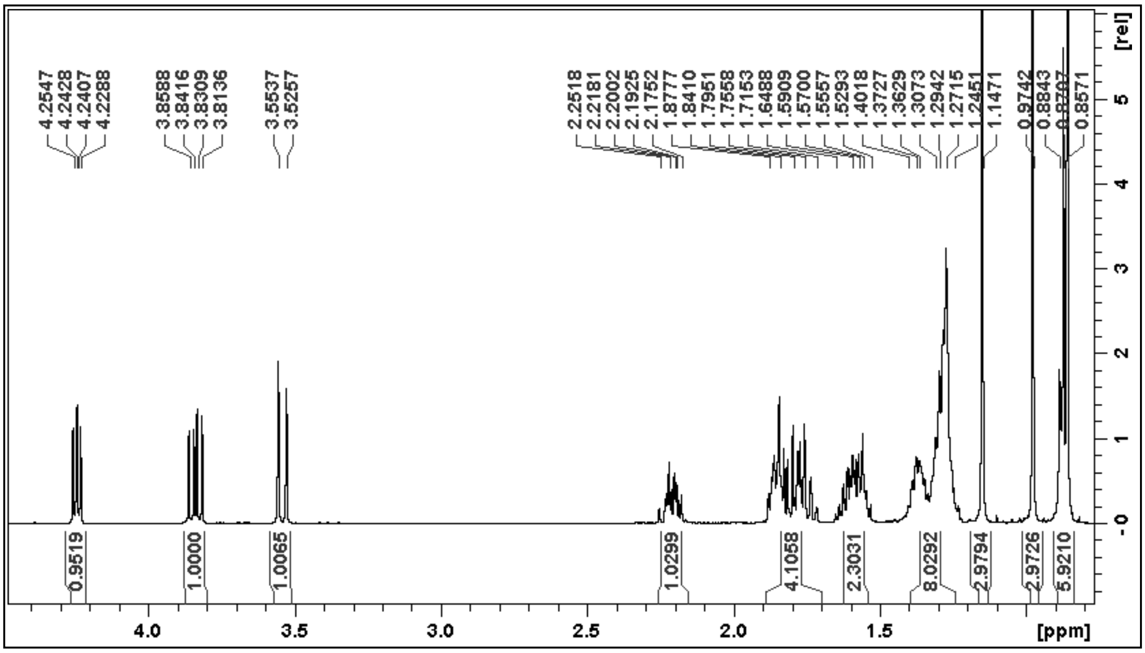

Figure S27. ^{1}H NMR spectrum of compound **1**

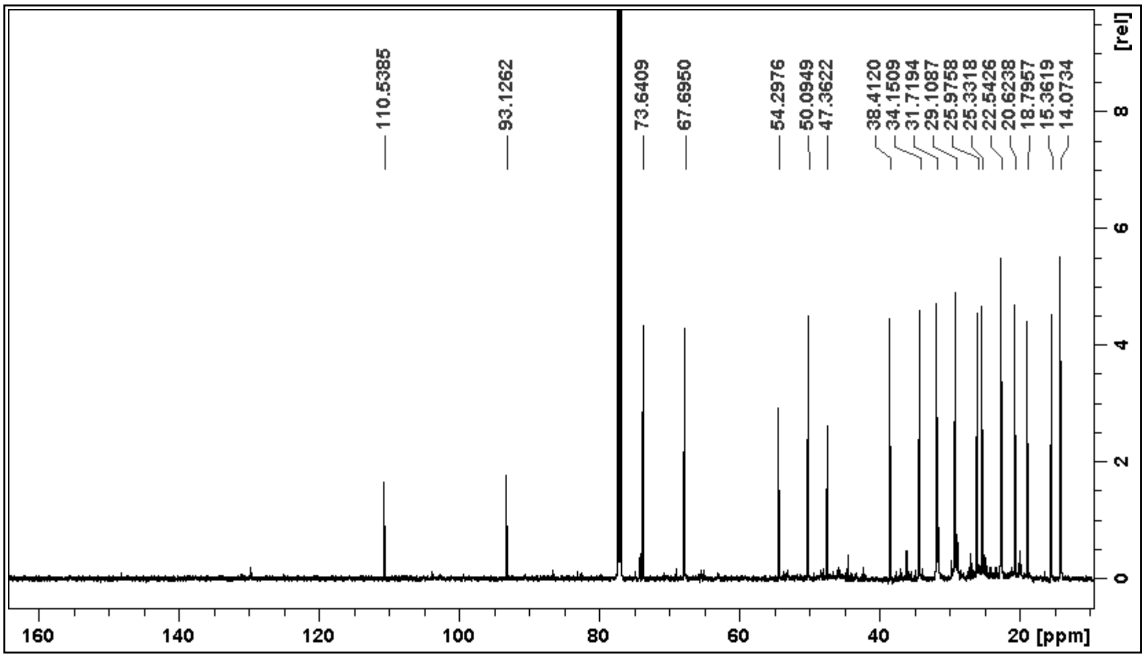

Figure S28. ^{13}C NMR spectrum of compound **1**

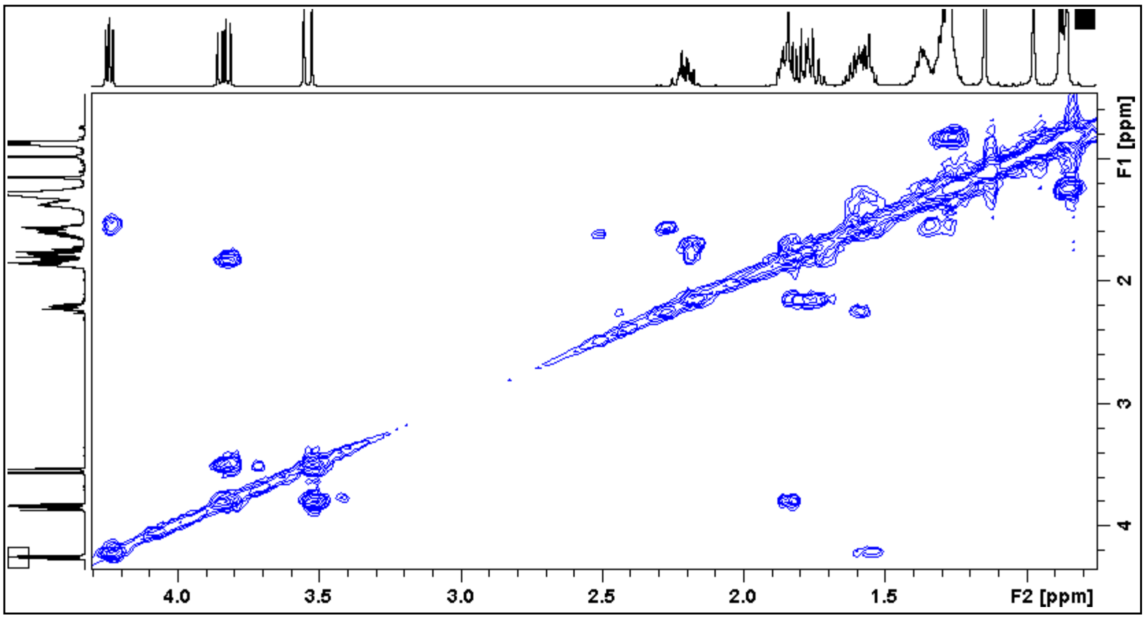

Figure S29. NMR-COSY spectrum of compound **1**

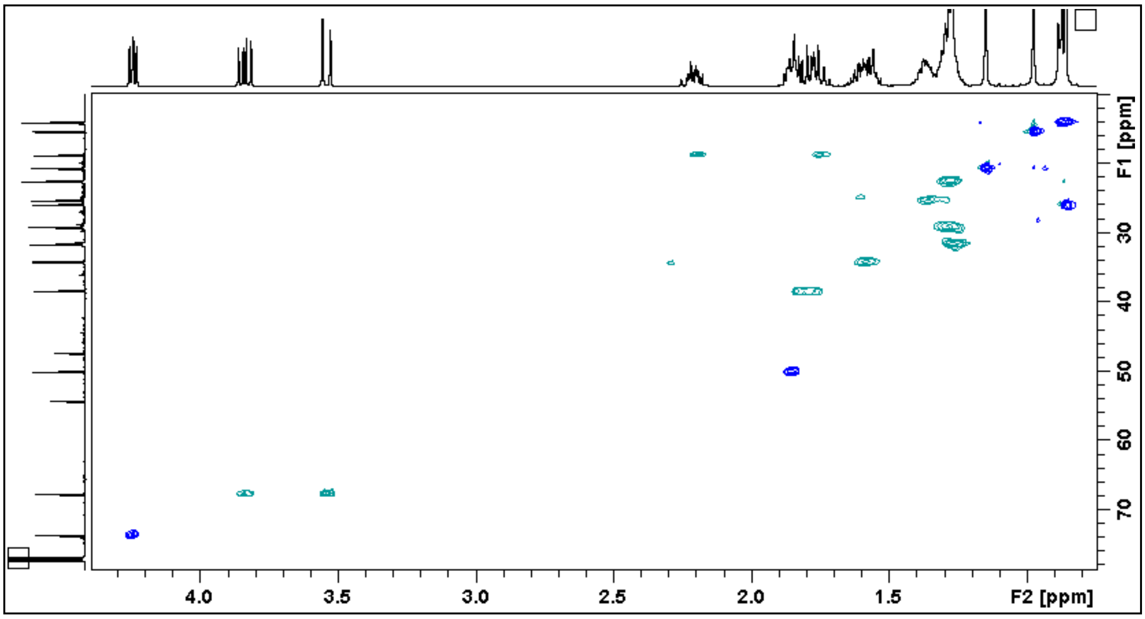

Figure S30. NMR-HSQC spectrum of compound **1**

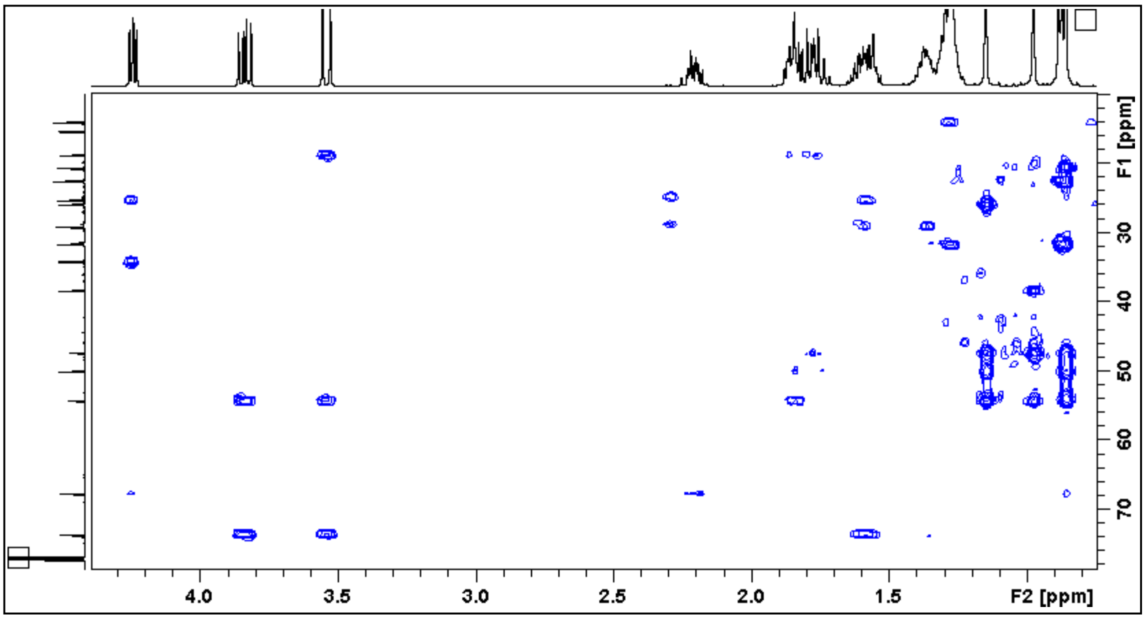

Figure S31. NMR-HMBC spectrum of compound **1**

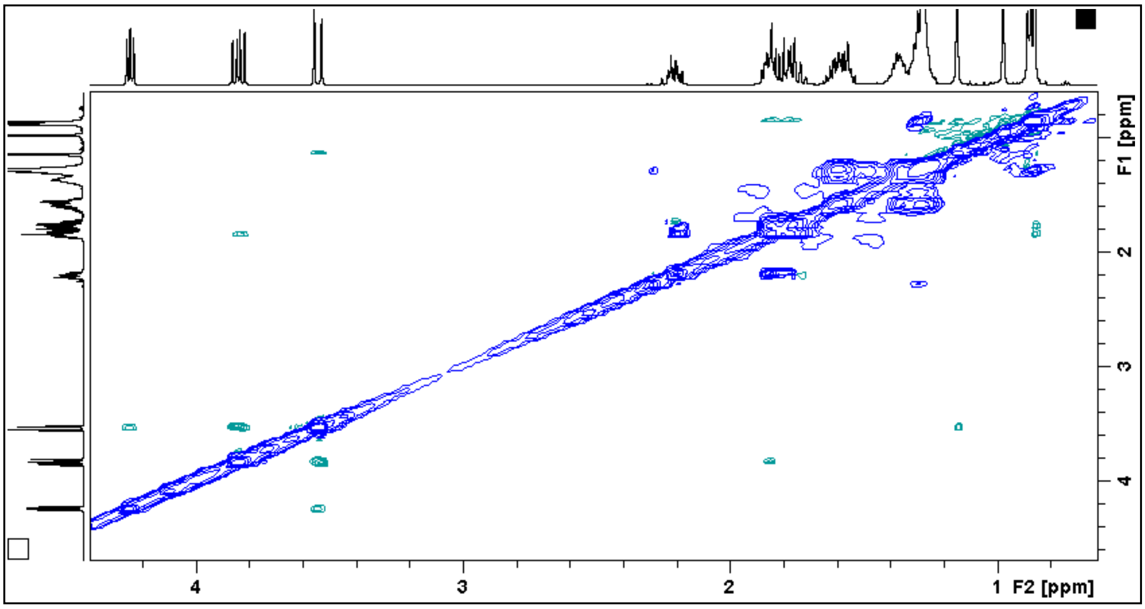

Figure S32. NMR-ROESY spectrum of compound **1**

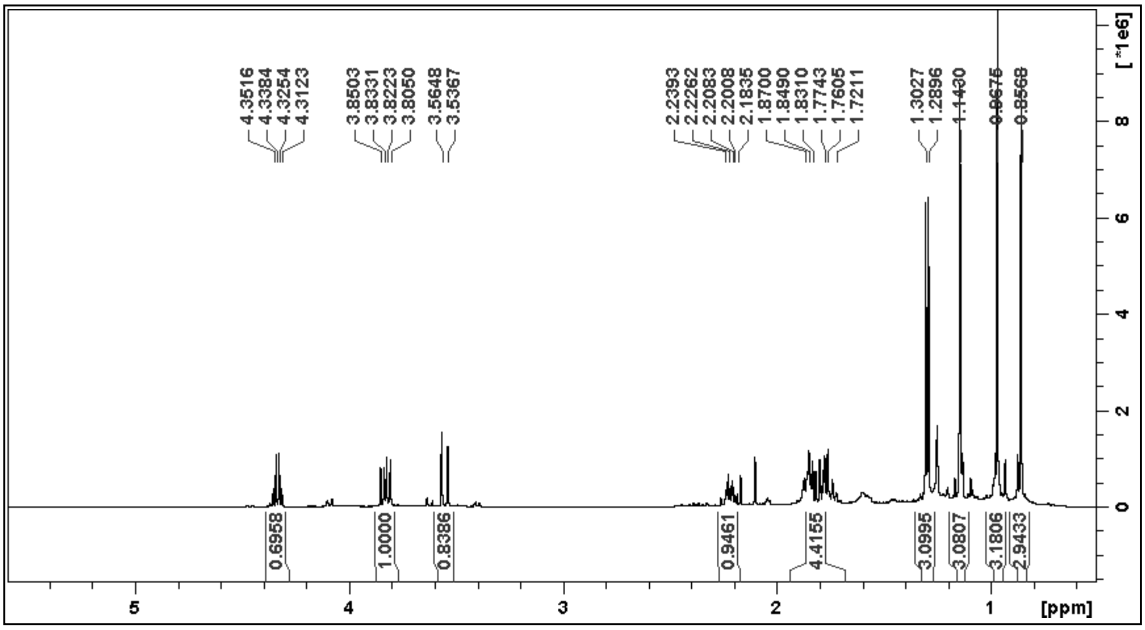

Figure S33. ^{1}H NMR spectrum of compound **21**

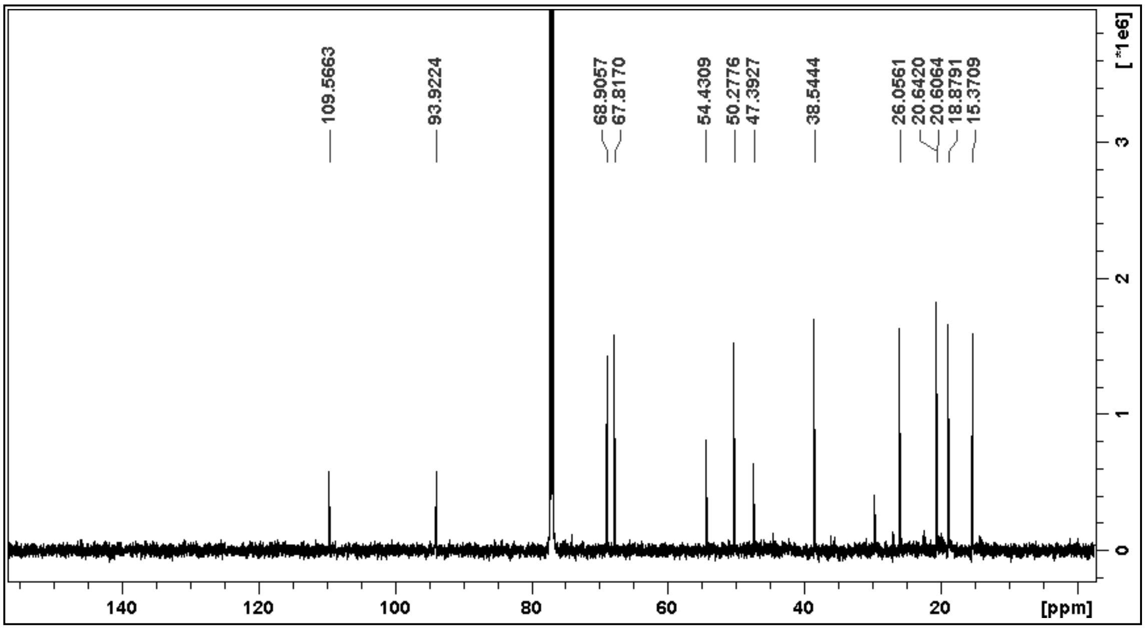

Figure S34. ^{13}C NMR spectrum of compound **21**

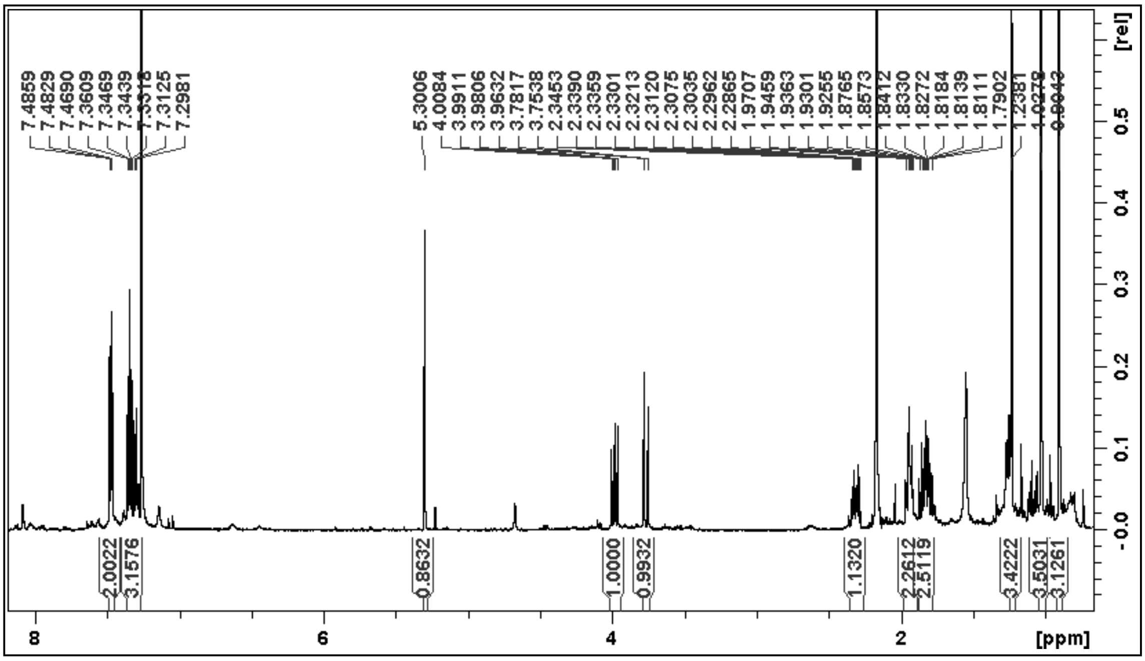

Figure S35. ^{1}H NMR spectrum of compound **22**

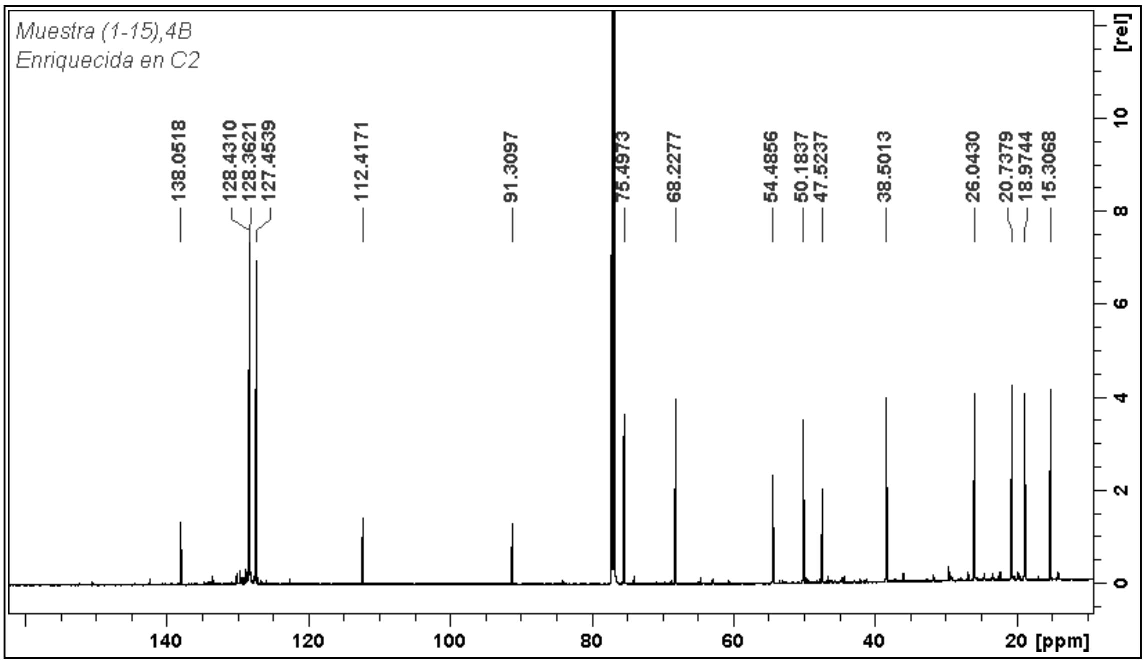

Figure S36. ^{13}C NMR spectrum of compound **22**

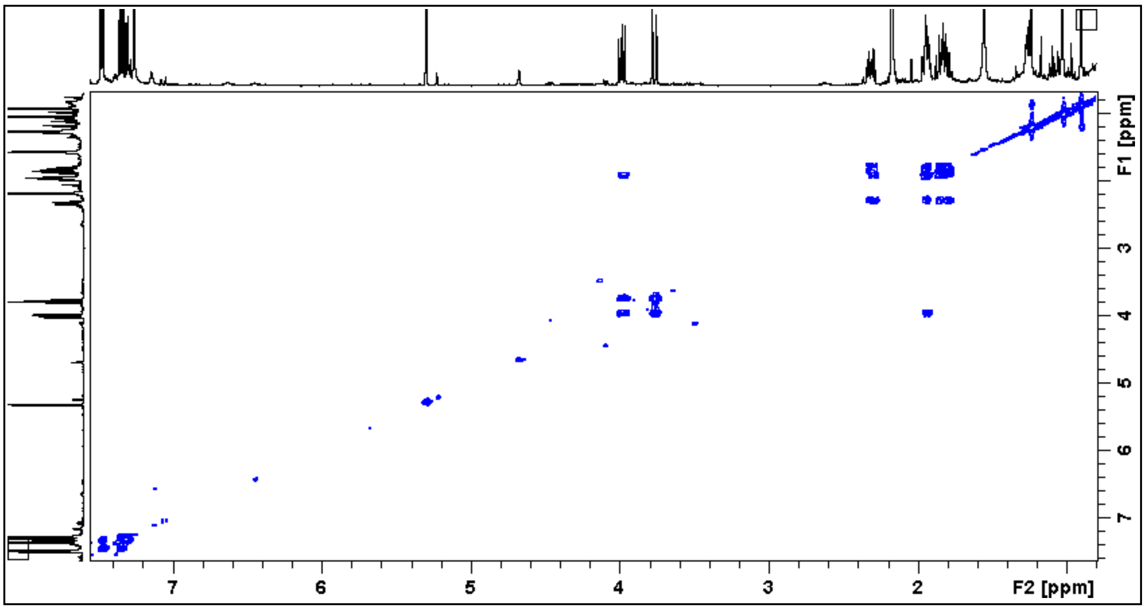

Figure S37. NMR-COSY spectrum of compound **22**

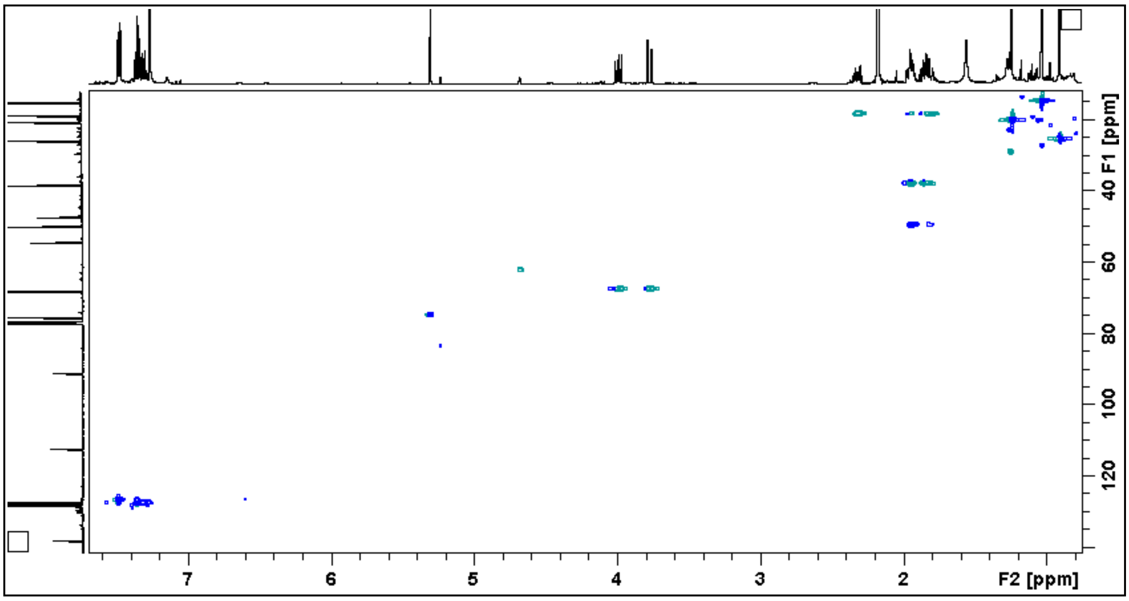

Figure S38. NMR-HSQC spectrum of compound **22**

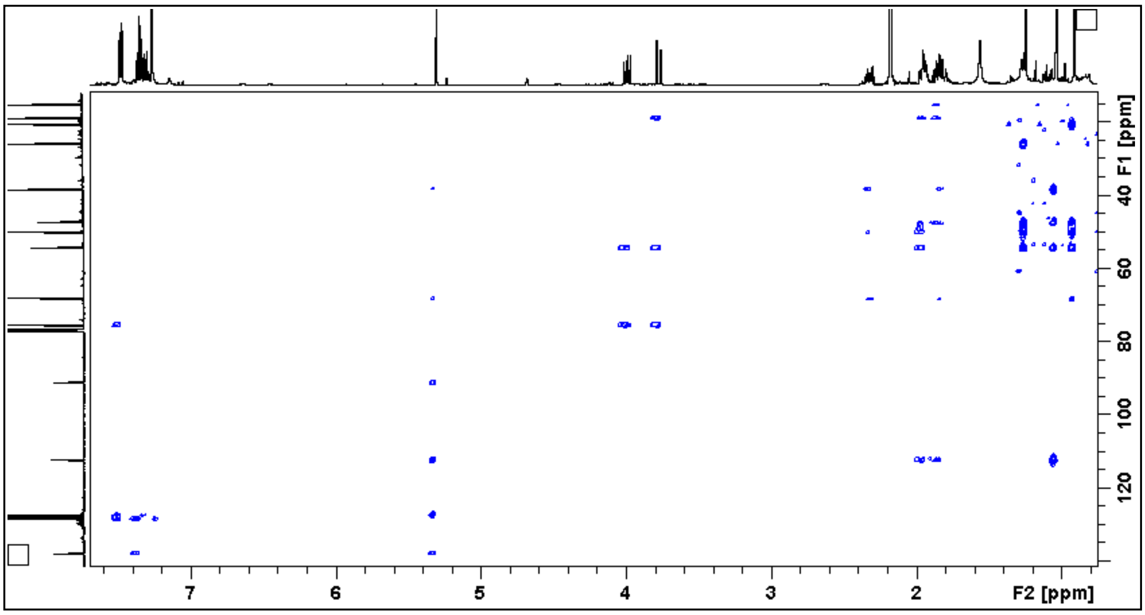

Figure S39. NMR-HMBC spectrum of compound **22**

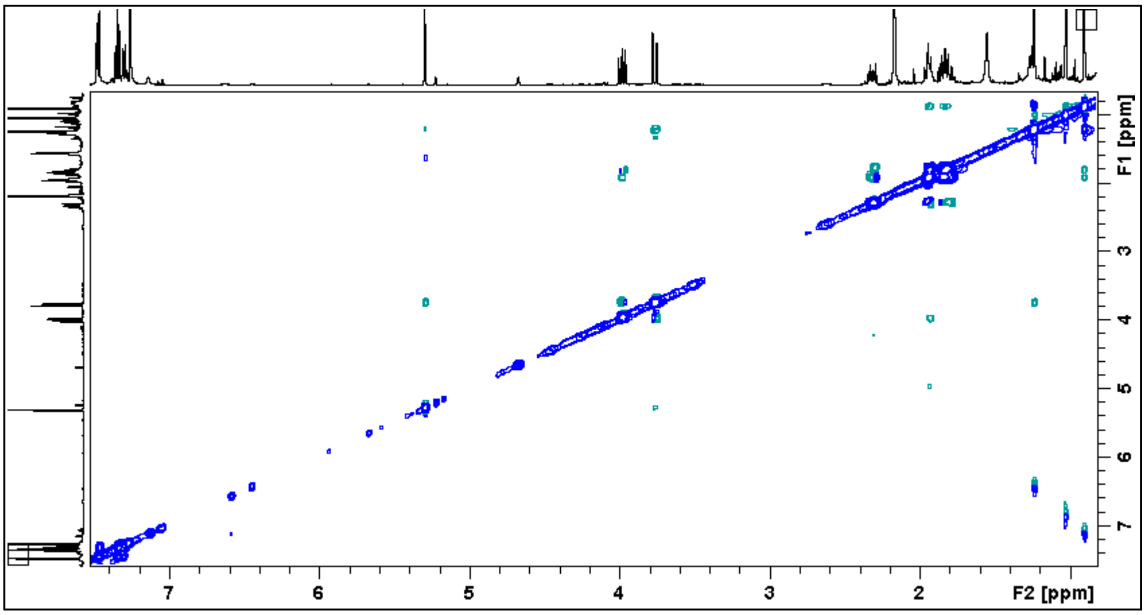

Figure S40. NMR-ROESY spectrum of compound **22**

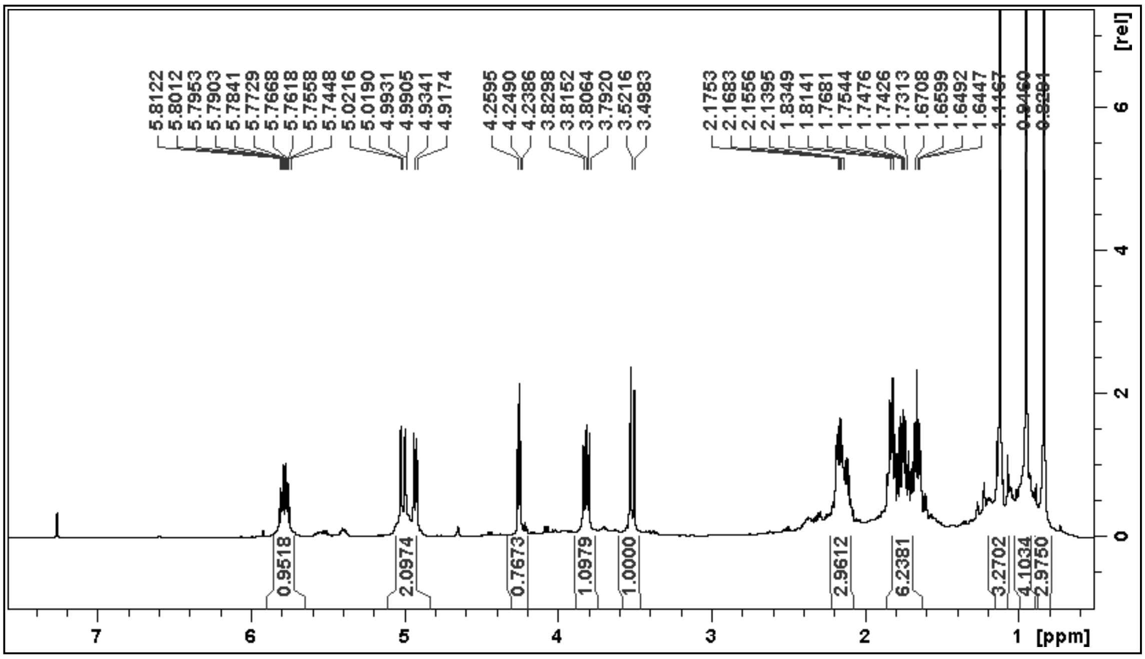

Figure S41. ^{1}H NMR spectrum of compound **23**

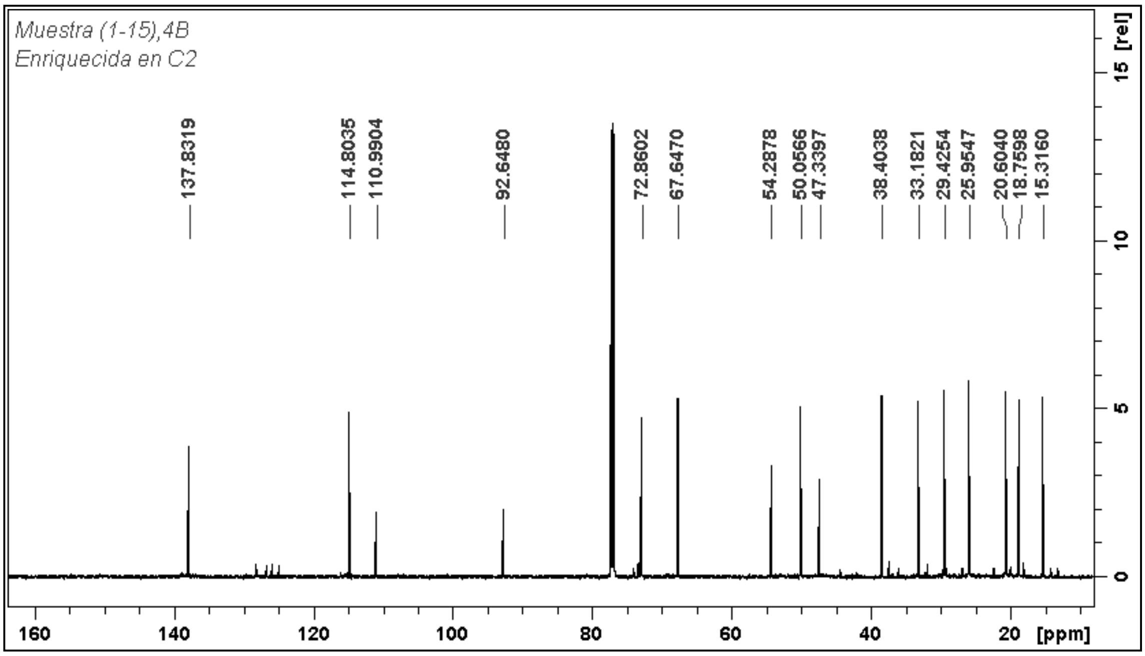

Figure S42. ^{13}C NMR spectrum of compound **23**

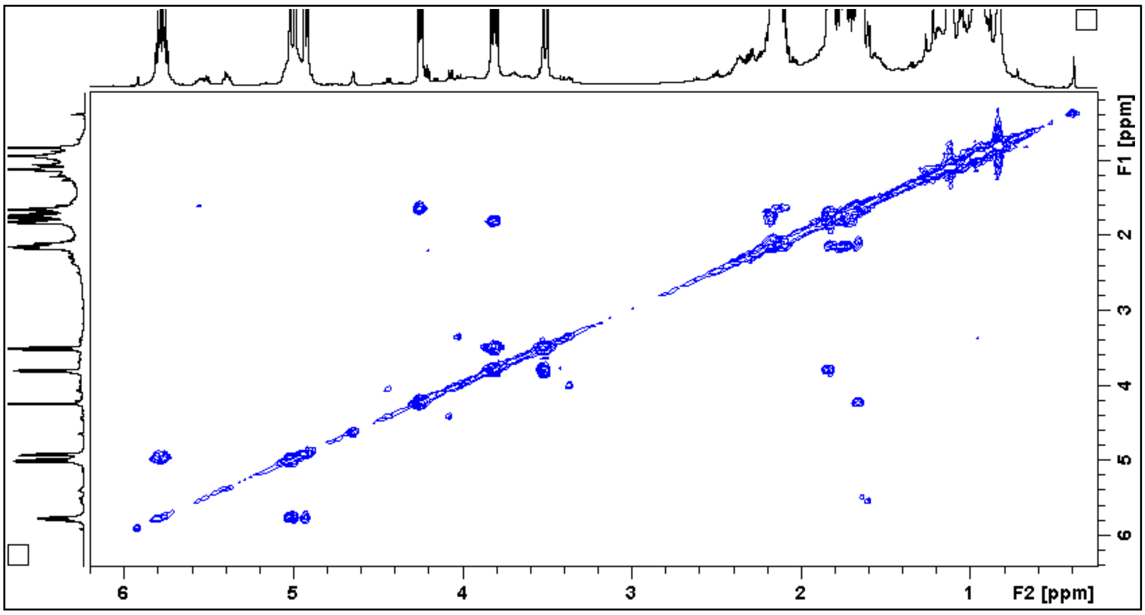

Figure S43. NMR-COSY spectrum of compound **23**

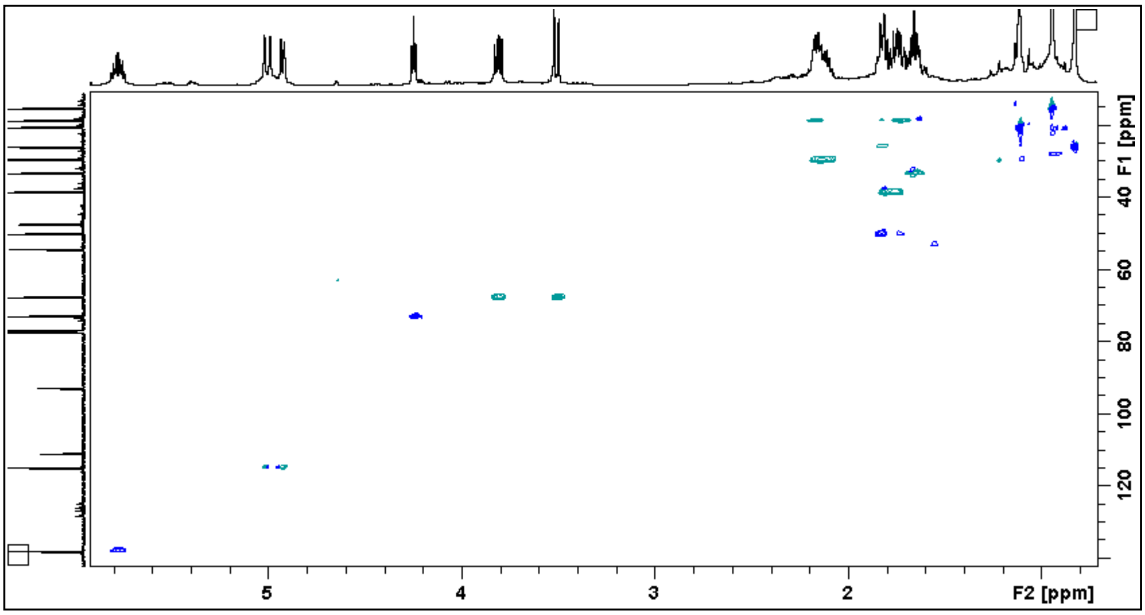

Figure S44. NMR-HSQC spectrum of compound **23**

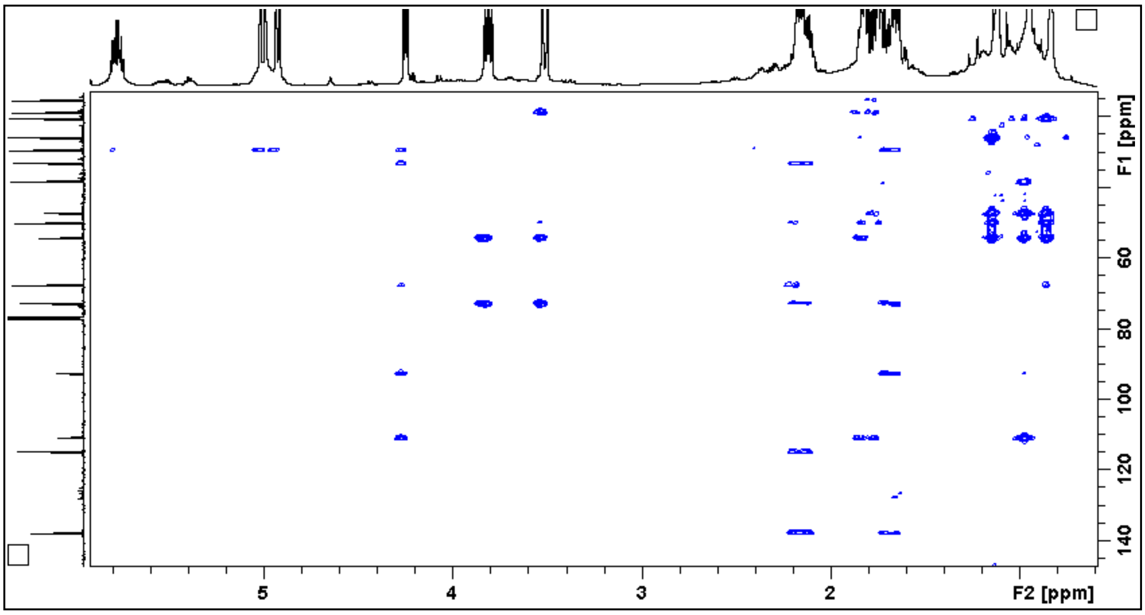

Figure S45. NMR-HMBC spectrum of compound **23**

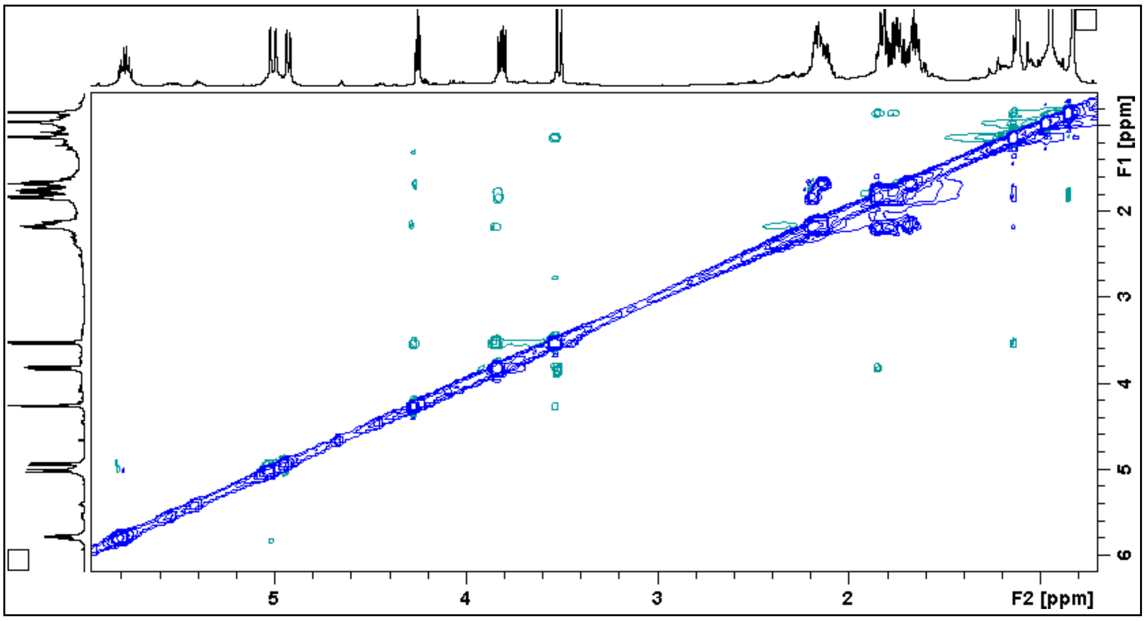

Figure S46. NMR-ROESY spectrum of compound **23**

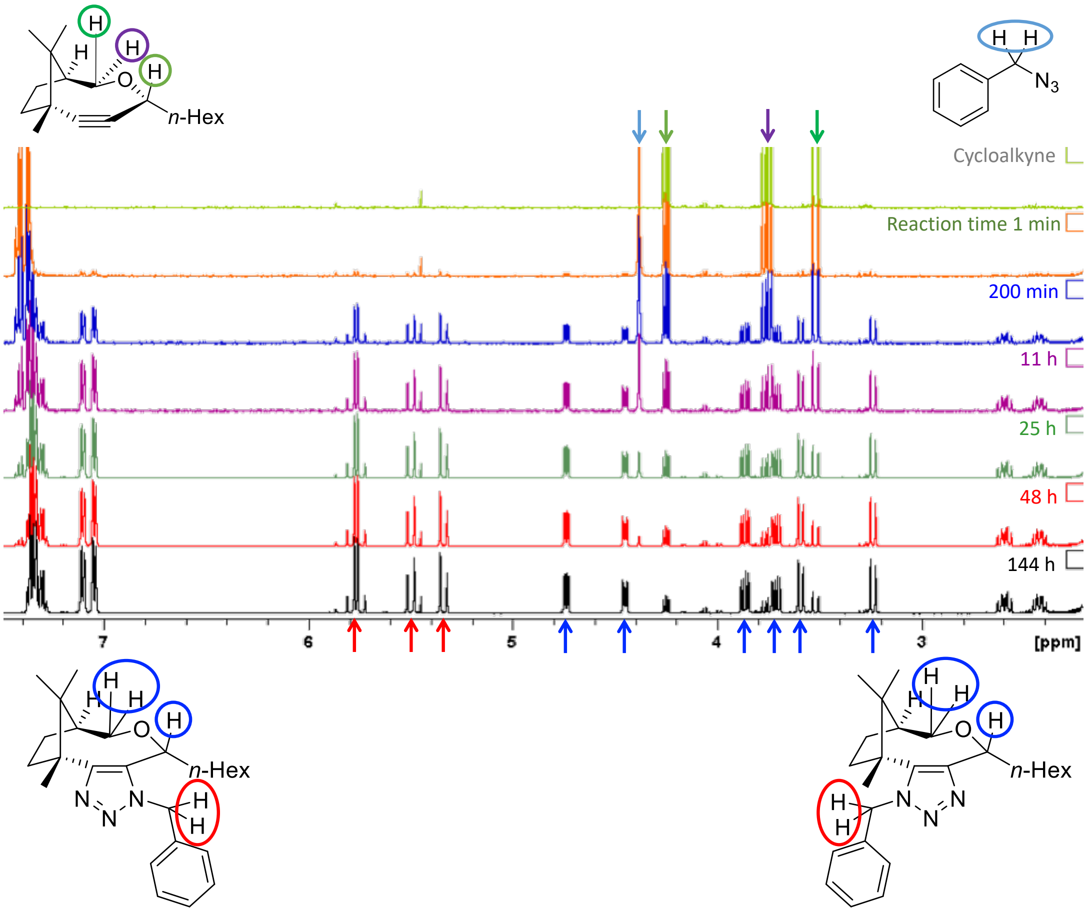

Figure S47. Monitoring SPAAC of cyclooctyne **1** and benzylazide by ^{1}H NMR. The signals of the most relevant protons of each structure are indicated with colored arrows in the spectra, which match with the colored circles drawn on the structures

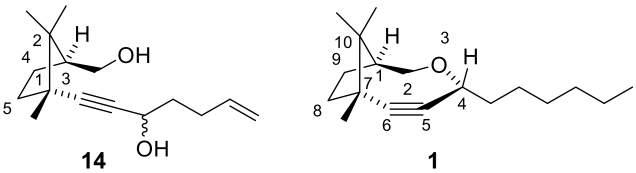

Figure S48. Numeration of atoms used for the nomenclature of acyclic precursors (e.g. compound **14**) and cyclic products (e.g. compound **1**)
